# Cationic NHC‐Phosphine Iridium Complexes: Highly Active Catalysts for Base‐Free Hydrogenation of Ketones

**DOI:** 10.1002/chem.202002811

**Published:** 2020-09-17

**Authors:** Xu Quan, Sutthichat Kerdphon, Bram B. C. Peters, Janjira Rujirawanich, Suppachai Krajangsri, Jira Jongcharoenkamol, Pher G. Andersson

**Affiliations:** ^1^ Department of Organic Chemistry Stockholm University Stockholm 10691 Sweden; ^2^ School of Chemistry and Physics University of Kwazulu-Natal Private Bag X54001 Durban 4000 South Africa

**Keywords:** asymmetric hydrogenation, iridium catalysis, ketones, ligand development, N-heterocyclic carbene

## Abstract

Novel bidentate N‐heterocyclic carbene‐phosphine iridium complexes have been synthesized and evaluated in the hydrogenation of ketones. Reported catalytic systems require base additives and, if excluded, need elevated temperature or high pressure of hydrogen gas to achieve satisfactory reactivity. The developed catalysts showed extremely high reactivity and good enantioselectivity under base‐free and mild conditions. In the presence of 1 mol % catalyst under 1 bar hydrogen pressure at room temperature, hydrogenation was complete in 30 minutes giving up to 96 % *ee*. Again, this high reactivity was achieved in additive‐free conditions. Mechanistic experiments demonstrated that balloon pressure of hydrogen was sufficient to form the activate species by reducing and eliminating the 1,5‐cyclooctadiene ligand. The pre‐activated catalyst was able to hydrogenate acetophenone with 89 % conversion in 5 min.

## Graphical abstract

**Base‐free**: New bidentate N‐heterocyclic carbene‐phosphine iridium complexes have been synthesized and evaluated in the hydrogenation of ketones. The majority of previously reported catalytic systems require base additives and if excluded, need elevated temperature or high pressure of hydrogen gas to achieve satisfactory reactivity. The developed catalysts showed high reactivity and good enantioselectivity (up to 96% *ee*) under additive‐free and mild conditions (balloon pressure H_2_, room temperature, 30 min).

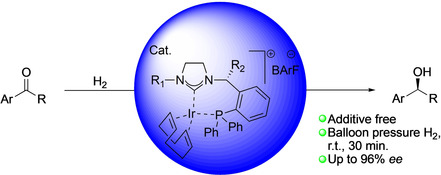

## Introduction

Chiral alcohol motifs represent a frequently occurring functional group in natural products or their intermediates.^1^ Among the methods to synthesize optically pure alcohols, transition‐metal‐catalyzed asymmetric hydrogenation of ketones represents one of the most efficient strategy in terms of reactivity, selectivity and atom‐economy. In 1995, Noyori described the use of a revolutionarily efficient [RuCl_2_(BINAP)‐(diamine)] catalyst for the asymmetric transfer hydrogenation of ketones.^2^ Encouraged by this development, considerable work has followed on the design of new diphosphine/diamine ligands and the evaluation of their corresponding Ru‐complexes, as well as elucidation of the reaction mechanism.^3^ In the case of iridium, successful ketone hydrogenations have been achieved by employing analogous conditions to the ruthenium systems in combination with a number of chiral diamine and diphosphine ligands.3d, 4 Although asymmetric hydrogenation of ketones is broadly utilized, the majority of these processes is base‐ or additive‐mediated. Limited examples of iridium catalyzed asymmetric ketone hydrogenation under neutral conditions have been developed.^5^ Despite the exclusion of base in these reactions, other limitations occur such as the use of elevated temperature, higher pressure of hydrogen or long reaction time. Therefore, the development of highly reactive catalysts that can hydrogenate ketones in the absence of additives and under mild conditions remains desired.

In the last decades, a vast study has been carried out on the preparation and implementation of N‐heterocyclic carbenes (NHCs) in organometallic chemistry.^6^ Due to their strong binding to metals, good stability and steric properties,^7^ NHCs offer a possibility to mimic phosphine ligands and numerous have successfully found applications in transition‐metal‐catalyzed reactions.^8^ The field of iridium‐catalyzed asymmetric hydrogenation faced an increase in the use of NHC ligands as well, with various successful applications being reported.8c, 9 Among these, bidentate imidazolylidene‐phosphine and imidazolinylidene‐phosphine ligands have been developed, mainly for the use in olefin hydrogenation.^10^ Ketones could also be reduced by means of ligated NHCs, however similar trends in reaction conditions are reported compared to classical phosphine ligands.3d, 4c, 8c, 11 Namely, additives are often required and if excluded, harsher conditions were applied. Herein, we report the development of highly active bidentate NHC‐phosphine‐Ir catalysts for the asymmetric hydrogenation of ketones under neutral conditions. These catalysts require very low H_2_ pressure and short reaction time without addition of base or acid.

## Results and Discussion

**Scheme 1:**
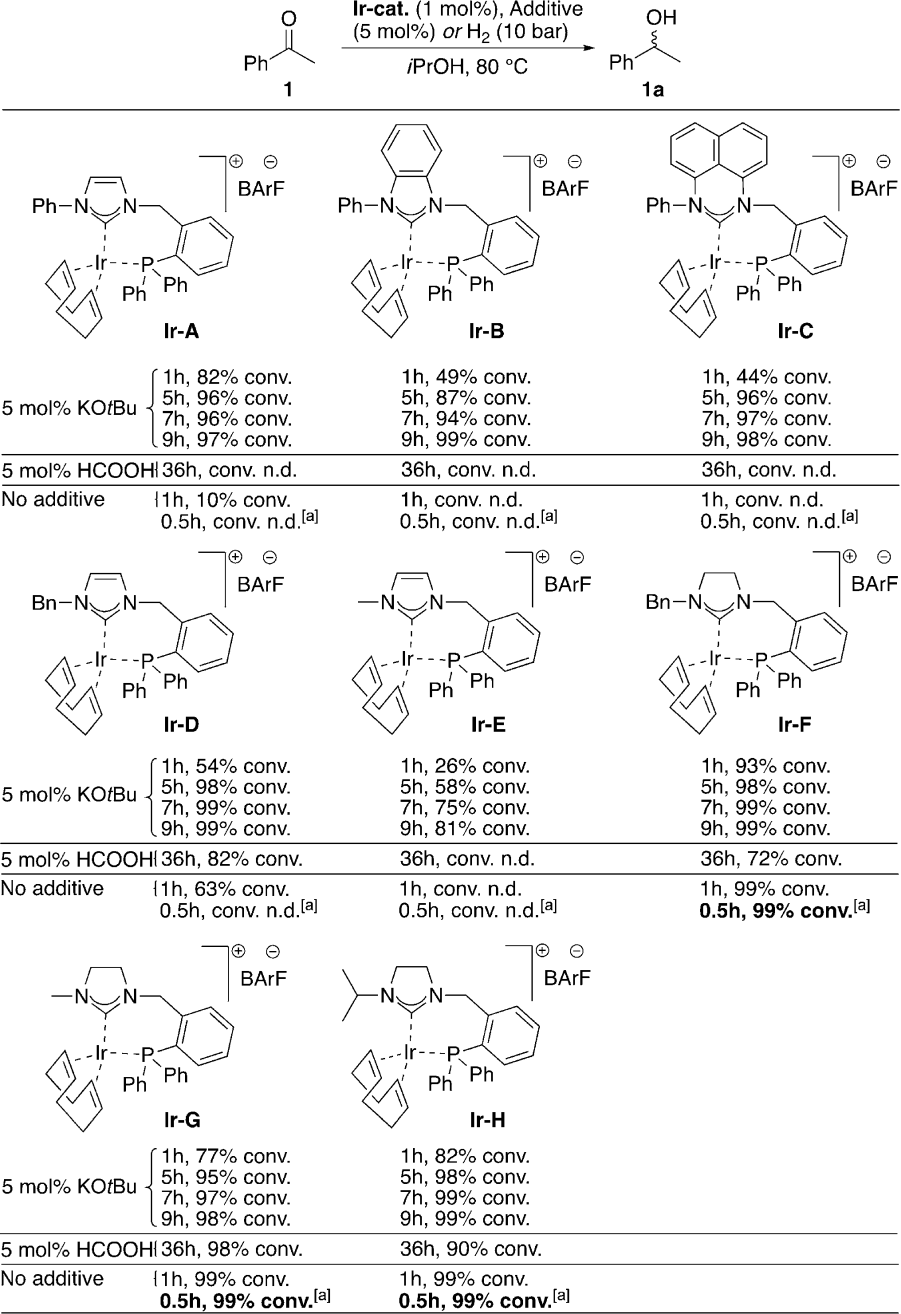
Achiral catalyst screening under basic, acidic and neutral conditions. Reaction conditions: 0.1 mmol **1**, 1.0 mol % catalyst, 5 mol % KO*t*Bu/HCOOH or H_2_ (10 bar), 2 mL *i*PrOH, 80 °C. a) H_2_ (balloon), 30 min, r.t.

First, several achiral NHC‐phosphine iridium complexes were prepared and evaluated in the reduction of acetophenone **1** under different reaction conditions (Scheme 1). Under basic reaction conditions (5 mol % of KO*t*Bu), all complexes showed good reactivity, and in most cases the reactions were complete in 9 h. Comparing the hydrogenation conversion in the first hour showed that iridium catalysts **Ir‐F** to **Ir‐H** with imidazolinylidene moieties were much more reactive than catalysts **Ir‐A** to **Ir‐E** bearing an imidazolylidene backbone. For example, in the first hour 54 % conversion was observed with **Ir‐D**, while for imidazolinylidene equivalent **Ir‐F** the reaction was almost complete with 93 % conversion. Under acidic conditions (5 mol % of formic acid), reactions were much slower and the catalytic activity strongly depended on the ligand structure. Over 36 hours, no conversion was observed for catalysts **Ir‐A** to **Ir‐C** and **Ir‐E**, while iridium catalysts **Ir‐F** to **Ir‐H** performed well and gave 72 %, 98 % and 90 % conversions, respectively. Next, the iridium catalysts were tested under hydrogen pressure without any added base or acid. The reactivity of the catalysts still depended strongly on the ligand structure. Catalysts with imidazolylidene‐derived ligand (**Ir‐A** to **Ir‐E**) were not or minor reactive under 10 bar of H_2_ and 80 °C. Only **Ir‐A** (10 % conversion) and **Ir‐D** (63 % conversion) were found to be reactive under these conditions. However, full conversions were achieved with **Ir‐F** to **Ir‐H** in 1 h. Gratifyingly, iridium catalysts **Ir‐F** to **Ir‐H** could retain their high reactivity under balloon pressure of H_2_ at room temperature, affording completion of the reaction in 30 min. These results are noteworthy since iridium‐catalyzed hydrogenation of ketones using H_2_ typically requires harsher conditions either in terms of higher pressure or elevated temperature.

**Scheme 2:**
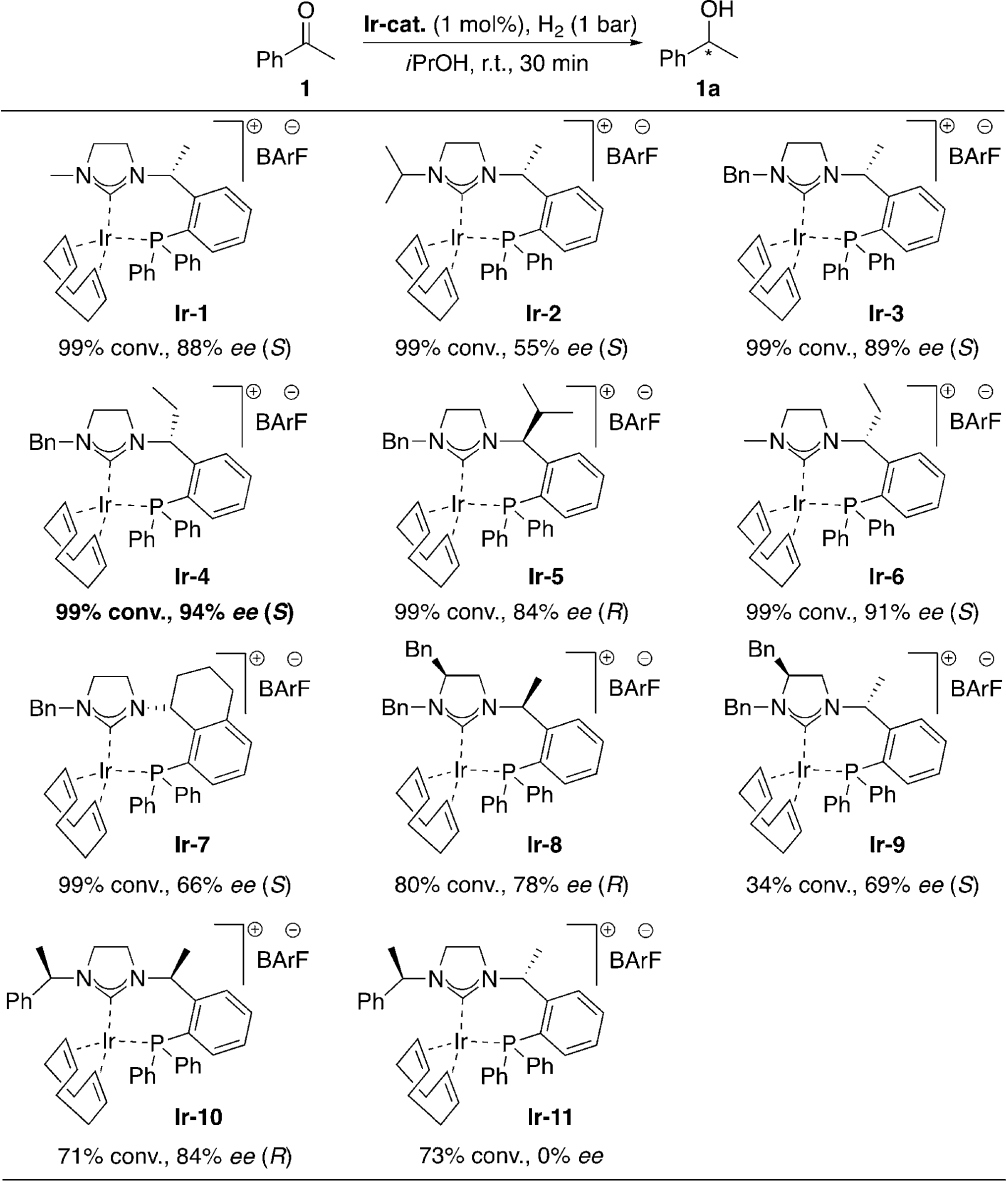
Chiral catalyst screening under neutral conditions. Reaction conditions: 0.1 mmol **1**, 1.0 mol % catalyst, H_2_ (1 bar), 2 mL *i*PrOH, 30 min, r.t.

It was encouraging that bidentate iridium imidazolinylidene‐phosphine complexes were able to catalyze the hydrogenation of ketones under such mild and additive‐free conditions. We then focused on the development of a stereoselective version of the reaction. Thus, chiral imidazolinylidene‐phosphine iridium catalysts **Ir‐1** to **Ir‐11** were synthesized and evaluated in the enantioselective hydrogenation of acetophenone **1** (Scheme 2).

Initially, the effect of solvent, temperature and pressure on the enantioselectivity was studied using **Ir‐3** and **Ir‐4** before evaluation of several chiral catalysts **Ir‐1** to **Ir‐11** (see Supporting Information). A solvent screening showed that the cleanest product formation accompanied with the highest enantioselectivity was obtained in *i*PrOH. Then, significant enhancement of the *ee* was observed upon decreasing the temperature, which increased from 70 % *ee* at 80 °C to 94 % *ee* at temperatures <40 °C. The reaction was surprisingly independent to hydrogen pressure and balloon pressure of H_2_ was sufficient to retain high conversion and enantioselectivity (full conversion, 94 % *ee*) for **Ir‐4**. The evaluation of catalysts **Ir‐1** to **Ir‐11** was thus performed using 1.0 mol % of catalyst under 1 bar H_2_ pressure in *i*PrOH, which in most cases gave complete consumption of starting material in 30 minutes at room temperature (Scheme 2). Complex **Ir‐1** gave 99 % conversion with 88 % *ee*. When the methyl group on the nitrogen atom in **Ir‐1** was changed to 2‐propyl, the reactivity remained high, but a dramatic loss of enantioselectivity was observed (**Ir‐2**, 55 % *ee*). On the other hand, benzyl substituted complex **Ir‐3** had similar reactivity and enantioselectivity compared to **Ir‐1**, 99 % conversion and 89 % *ee*. A small increase in the bulkiness on the chiral carbon, methyl to ethyl (**Ir‐4**), enhanced the enantioselectivity to 94 % *ee*. No further improvement in the *ee* was observed when a more sterically demanding 2‐propyl substituent was introduced on the chiral carbon (**Ir‐5**, 84 % *ee*) or by replacing the benzyl group on nitrogen with a methyl group (**Ir‐6**, 91 % *ee*). Complex **Ir‐7**, derived from a chiral cyclic amine derivative, gave only 66 % *ee*.

Further modification of the ligand structure by installing an additional stereogenic center gave two pairs of diastereomeric iridium complexes **Ir‐8**/**Ir‐9** and **Ir‐10**/**Ir‐11**. The second stereogenic center on the imidazolinylidene moiety in **Ir‐8** and **Ir‐9** had no beneficial effect on the enantioselectivity and gave lower selectivity compared to **Ir‐3** (78 and 69 % *ee*, respectively). It is noteworthy that the configuration of the major formed enantiomer switched upon inversion of the second chiral center. Diastereomeric complexes **Ir‐10** and **Ir‐11** had comparable reactivity but differed greatly in their enantioselectivity (**Ir‐10** 84 % and **Ir‐11** 0 % *ee*). These results suggest that the enantioselectivity relies mainly on a combination of the steric properties on both sides of the N‐heterocyclic carbene.

The reactivity and properties of these novel catalysts were further studied. A kinetic study in the early stage of the hydrogenation using **Ir‐2**, **Ir‐4** and **Ir‐8** showed that the highest initial turnover frequency was obtained using **Ir‐4**, giving 19 % conversion in one minute of reaction time (Table 1). The conversion gradually increased over the course of 20 min resulting in 45 % conversion in 5 min and 98 % conversion after 16 min. Catalyst **Ir‐2** showed a lower, but still high, reaction rate with 16 % conversion after 5 min and completion of the reaction after 20 min. Catalyst **Ir‐8** was found to be less reactive obtaining less than 1 % of product in 2 min and only 3 % after 5 min.

**Table 1:** Conversion of acetophenone over time and the effect of pre‐activated catalyst.

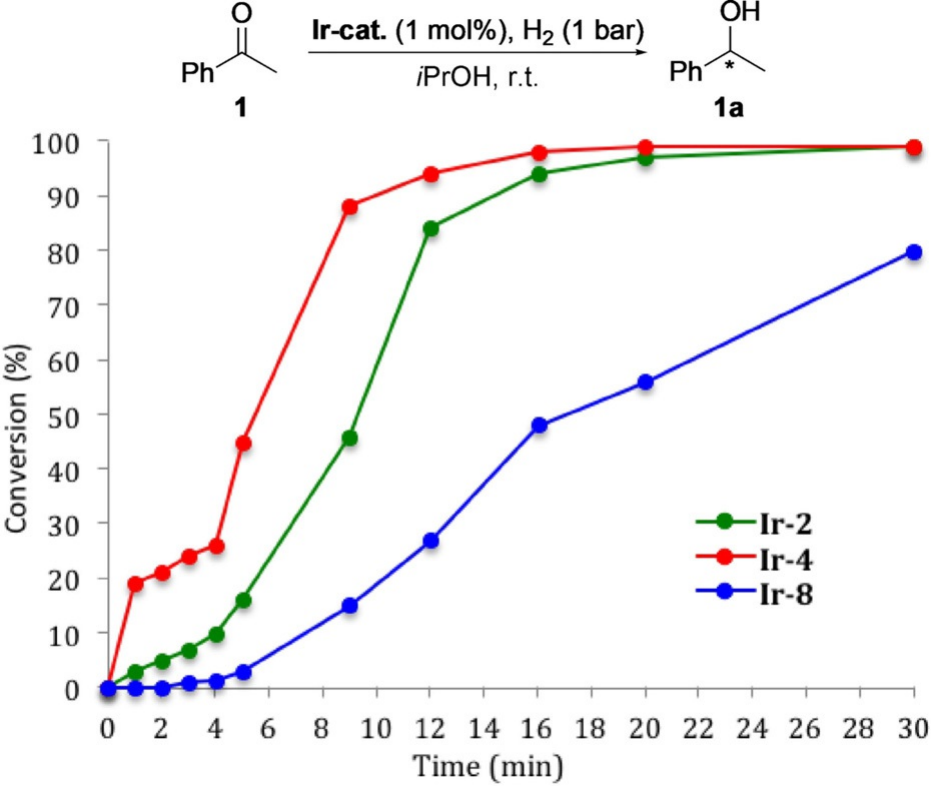				
Catalyst	Reaction conditions	Time	conv.	*ee*
**Ir‐2**	pre‐activated^[a]^	5 min	65 %	55 % (*S*)
**Ir‐2**	standard conditions	5 min	16 %	55 % (*S*)
**Ir‐4**	pre‐activated^[a]^	5 min	89 %	94 % (*S*)
**Ir‐4**	standard conditions	5 min	45 %	94 % (*S*)
**Ir‐8**	pre‐activated^[a]^	5 min	26 %	78 % (*R*)
**Ir‐8**	standard conditions	5 min	3 %	–

We then attempted to pre‐activate the catalyst with hydrogen gas before addition of substrate **1**. The enantioselectivity remained unchanged, while the conversions increased significantly. Acetophenone **1** was hydrogenated to reach 65 %, 89 % and 26 % conversion within 5 min when using the pre‐activated catalysts of **Ir‐2**, **Ir‐4** and **Ir‐8**, compared to 16 %, 45 % and 3 % conversion under standard conditions, respectively.

In order to shed further light on the nature of the active catalyst, a reaction using 10 mol % of **Ir‐4** was analyzed after completion and showed that over 80 % of the theoretical amount of cyclooctane was generated under balloon pressure of H_2_ in 30 min. Moreover, 1 equiv of **Ir‐4** and 2 equiv of **1** together with 5 equiv of *i*PrOH were reacted in *d*_8_‐THF (balloon pressure of H_2_, 1 h) and >95 % of the theoretical amount of cyclooctane was observed by ^1^H NMR analysis. To this reaction mixture was then added 100 equiv of **1**, which was hydrogenated in 79 % in 30 min. These combined results clearly indicate that during the pre‐activating period, the COD ligand is hydrogenated into cyclooctane, and this results in the formation of the active hydrogenation catalyst.

**Scheme 3:**
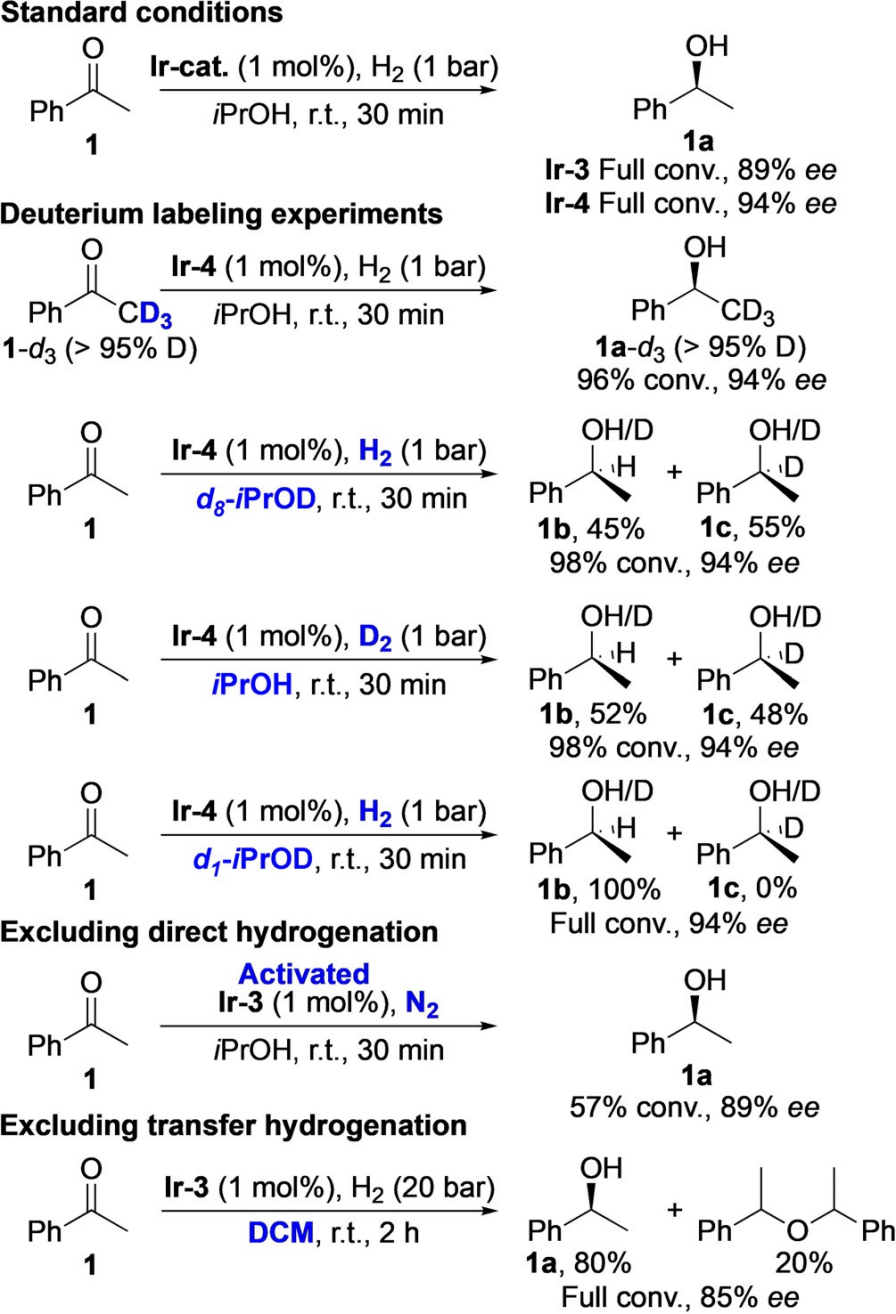
Mechanistic studies.

**Scheme 4:**
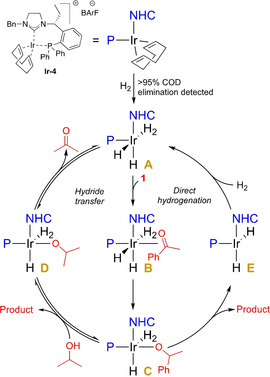
Proposed catalytic pathways.

Thereafter, the mechanism of the hydrogenation was studied (Scheme 3). **1**‐*d*_3_ was prepared and subjected to standard conditions without the loss of any deuterium in α‐position, indicating that the reaction does not involve the hydrogenation of an enol. By using *d*_8_‐*i*PrOD as the solvent and employing hydrogen gas, a mixture of products was obtained with a deuterium incorporation of 55 % on the carbinol carbon **1 c**. A similar amount of 48 % **1 c** was obtained when deuterium gas in *i*PrOH was used. To verify that the formation of a mixture of **1 b**/**1 c** did not arise from a hydride exchange between any of the Ir‐hydride intermediates and the solvent, *d*_1_‐*i*PrOD was used as a solvent hydrogenating acetophenone **1** to **1 b** in a clean manner. Since our developed ligand structures lack a heteroatom that can act as a proton acceptor, a transfer hydrogenation pathway where in a single step the proton and the hydride are abstracted from the solvent is unlikely. However, stepwise pathways involving external delivery of the proton (i.e., from the solvent), after migratory insertion of a hydride from the Ir‐species, are reported for the hydrogenation of ketones using Ir‐NHC complexes.^12^ To test this option, where hydrogen gas is not involved, activated **Ir‐3** reacted with **1** in *i*PrOH under N_2_ atmosphere giving 57 % conversion in 30 min without any loss of selectivity (89 % *ee*). Additionally, from the solvent screening, 41 % and full conversion was observed when the reaction was performed in toluene and DCM, respectively (**Ir‐3**, 20 bar of H_2_, see Supporting Information), and therefore a direct hydrogenation mechanism cannot be excluded. Based on these results, we propose two coexisting active hydrogenation mechanisms which pathways are given in Scheme 4. Starting from **Ir‐4**, the pre‐catalyst gets activated under hydrogen atmosphere forming hydrogenation active dihydride species **A**. After coordination of the substrate (intermediate **B**), migratory insertion of a hydride to the carbonyl carbon takes place to form **C** at which point the pathway can diverge. Following a hydride transfer pathway, the proton is delivered externally by the solvent and regeneration of the catalyst occurs by hydride abstraction of the formed Ir‐isopropoxide species **D**. Alternatively, the Ir‐alkoxide bond in **C** can be cleaved by the ligated dihydrogen forming **E** and subsequent coordination of a new molecular hydrogen regenerates **A**. The reversibility of these hydrogenation/dehydrogenation steps via **D** can possibly account for the H/D scrambling in the product when D_2_ gas or *d*_8_‐*i*PrOD is used. Interestingly, the selectivity in DCM (85 % *ee*) is close to the selectivity observed in *i*PrOH (89 % *ee*), what suggests a similar coordination of the substrate upon iridium regardless of the underwent pathway. The slightly lower value in DCM can be explained by the observed, reversible, ether formation.

**Scheme 5:**
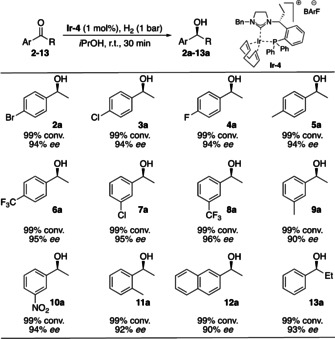
Substrate scope. Reaction conditions: 0.1 mmol **1**, 1.0 mol % catalyst, H_2_ (1 bar), 2 mL *i*PrOH, 30 min, r.t.

Finally, the substrate scope was investigated and the catalyst maintained high reactivity in most cases with the reaction being complete in 30 minutes (Scheme 5). The hydrogenation was successful for a wide range of substituted acetophenones. The nature of the *para*‐substituent showed not to interfere with the selectivity. Thus, both electron‐donating methyl, as well as various weakly to strong electron‐withdrawing groups were well tolerated giving 94–96 % *ee* (**2**–**6**). A similar trend was observed for *meta*‐substitutions giving access to 90–96 % *ee* with full conversion (**7**–**10**). Furthermore, *ortho*‐methyl substituted acetophone **11** resulted in 92 % *ee* and naphthyl ketone **12** was hydrogenated in 90 % *ee*. Prolonging the ketone side‐chain to ethyl (**13**) gave equal selectivity compared to **1** (93 % *ee*). Unfortunately, acetylated heteroaromatic substrates were found to inhibit the reaction.^13^

## Conclusions

In summary, the development of a new library of NHC‐phosphine iridium complexes was reported and their efficiency in the hydrogenation of ketones was demonstrated. On the basis of an initial screening of achiral ligand backbones, an asymmetric version of the reaction was designed and optimized, obtaining up to 96 % *ee* for acetophenone derivatives. The extent of chiral induction greatly depended on the substitution pattern on both sides of the imidazolinylidene moiety. Remarkably, these catalysts showed very high reactivity under balloon pressure of hydrogen in *i*PrOH, with most reactions being complete in 30 minutes. Upon pre‐activation of the most enantioselective catalyst, 89 % conversion could be obtained in only 5 min reaction time. Notably, these novel bidentate NHC‐phosphine catalysts performed well under additive‐free conditions, whereas a base is often required in the hydrogenation of ketones. During the activation period, COD is reduced and eliminated as cyclooctane. Relying on mechanistic experiments, a hydride transfer pathway as well as a direct hydrogenation pathway are proposed.

## Experimental Section

The synthetic procedures and characterization data of all new iridium complexes as well as their intermediates is given in the Supporting Information.

### Representative procedure for the hydrogenation of aryl ketones

An oven‐dried glass vial equipped with magnetic stirring bar was charged with substrate (0.1 mmol), iridium complex (1.0 mol %) and dry *i*PrOH (2.0 mL). The vial was placed in a low‐pressure hydrogenation apparatus, purged three times with nitrogen, purged three times with hydrogen gas and then pressurized to 1.0 bar with hydrogen gas. After stirring for 30 min at room temperature, the pressure was released and the solvent was removed under vacuum. The crude product was then purified by column chromatography on silica gel (pentane/Et_2_O, 1:1) to give the corresponding product. Conversions were determined by ^1^H NMR spectroscopy and the *ee* values were determined by GC or SFC analysis using chiral stationary phase. The configuration of the products was determined by comparing their sign of optical rotation with literature data.

## Conflict of interest

The authors declare no conflict of interest.

## Supporting information

As a service to our authors and readers, this journal provides supporting information supplied by the authors. Such materials are peer reviewed and may be re‐organized for online delivery, but are not copy‐edited or typeset. Technical support issues arising from supporting information (other than missing files) should be addressed to the authors.

Supplementary
