# Supplementary material for: Cationic NHC‐Phosphine Iridium Complexes: Highly Active Catalysts for Base‐Free Hydrogenation of Ketones
Source: Chemistry. 2020 Sep 17;26(58):13311–6. doi: 10.1002/chem.202002811 (PMC7693092; doi:10.1002/chem.202002811)
Supplement: Supplementary file 1 — Supplementary [file CHEM-26-13311-s001.pdf]

Chemistry–A European Journal

Supporting Information

Cationic NHC-Phosphine Iridium Complexes: Highly Active Catalysts for Base-Free Hydrogenation of Ketones

Xu Quan+,[a] Sutthichat Kerdphon+,[a] Bram B. C. Peters+,[a] Janjira Rujirawanich,[a] Suppachai Krajangsri,[a] Jira Jongcharoenkamol,[a] and Pher G. Andersson*[a, b]

Table of contents

General information……………………………………………………...………2

Supplementary tables……………………………………………………………….3

Solvent, pressure and temperature screening……………………………….3

Conversion kinetic study **Ir-2**, **Ir-4** and **Ir-8**……………………………….…4

General procedure for the hydrogenation of ketones……………………………….5

Procedure for the hydrogenation of acetophenone **1** using pre-activated catalyst…….5

Preparation of iridium complexes…………………………………………………..6

Preparation of achiral NHC-phosphine iridium complexes **Ir-A** to **Ir-H**…......6

Preparation of chiral NHC-phosphine iridium complexes **Ir-1** to **Ir-11**……...9

Separation of chiral products and chromatograms………………………………...29

References…………………………………………………………………………35

General information

Isopropanol was dried over calcium hydride, distilled and stored under nitrogen. Dichloromethane and toluene were dried over calcium hydride and freshly distilled under nitrogen. THF was distilled from sodium-benzophenone under nitrogen. The commercially available ketones were purified by either column chromatography or distillation. Thin-layer chromatography (TLC) was performed on aluminum plates coated with Kieselgel 60 (0.20 mm, UV 254) and visualized under ultraviolet light followed by staining with potassium permanganate solution. ^{1}H NMR spectra were recorded at 400 MHz in $CDCl_3$ and referenced internally to the residual $CHCl_3$ signal (7.26 ppm). ^{13}C NMR spectra were recorded at 100 MHz in $CDCl_3$ and referenced to the central peak of $CHCl_3$ (77.16 ppm). ^{31}P NMR spectra were recorded at 162 MHz in $CDCl_3$. Chemical shifts were reported in ppm (δ scale), and coupling constants (*J*) were reported in Hertz (Hz). IR spectra were obtained from Perkin-Elmer Spectrum one Spectrometer. High resolution mass spectrometric (HRMS) data were obtained from Bruker microTOF-Q II instrument operated at ambient temperatures.

Supplementary tables

Table S1. Solvent, pressure and temperature screening.

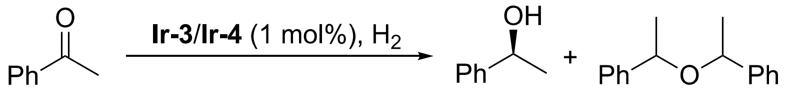

1 1a Ether by-product

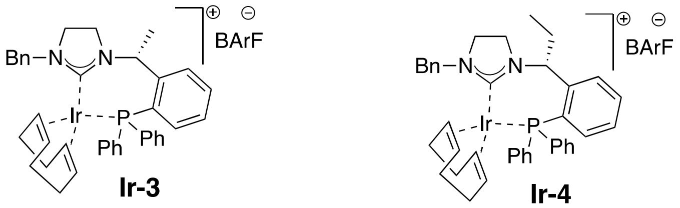

Ir-3 Ir-4

Entry	Catalyst	Solvent	Temperature	Time	H_2	Conversion[a]	*ee*
1	**Ir-3**	DCM	r.t.	2 h	20 bar	99% (20%)	85%
2	**Ir-3**	Tol	r.t.	4 h	20 bar	41% (8%)	53%
3	**Ir-3**	Et_2O	r.t.	4 h	20 bar	6%	-
4	**Ir-3**	DMSO	r.t.	4 h	20 bar	No	-
5	**Ir-3**	DMF	r.t.	4 h	20 bar	No	-
6	**Ir-3**	EtOH	r.t.	1 h	20 bar	8%	-
7	**Ir-3**	^{i}PrOH	r.t.	1 h	20 bar	99%	89%
8	**Ir-3**	^{i}PrOH	r.t.	30 min	Balloon	99%	89%
9	**Ir-4**	^{i}PrOH	80 °C	30 min	3 bar	99%	70%
10	**Ir-4**	^{i}PrOH	60 °C	30 min	3 bar	99%	75%
11	**Ir-4**	^{i}PrOH	40 °C	30 min	3 bar	99%	94%
12	**Ir-4**	^{i}PrOH	r.t.	30 min	3 bar	99%	94%
13	**Ir-4**	^{i}PrOH	10 °C	30 min	3 bar	97%	94%
14	**Ir-4**	^{i}PrOH	r.t.	30 min	1 bar	99%	94%
15	**Ir-4**	^{i}PrOH	r.t.	30 min	Balloon	99%	94%

Reaction conditions: 0.1 mmol **1**, 1.0 mol% catalyst, H_2, 2 ml solvent. a) Conversion to ether by-product is given in parenthesis.

Table S2. Conversions for the kinetic study using catalyst Ir-2, Ir-4 and Ir-8.

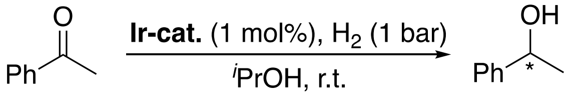

1 **1a**

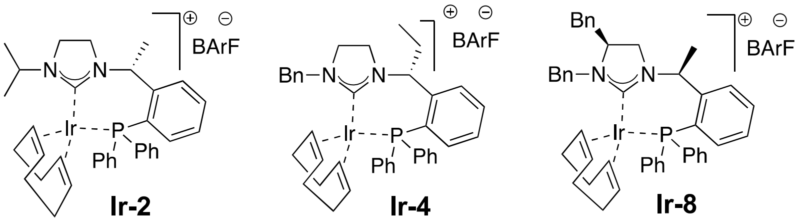

	**Ir-2**	**Ir-4**	**Ir-8**
**Reaction time**	**conversion**	**conversion**	**conversion**
1min	3%	19%	-
2min	5%	21%	-
3min	7%	24%	1%
4min	10%	26%	1.3%
5min	16%	45%	3%
9min	46%	88%	15%
12min	84%	94%	27%
16min	94%	98%	48%
20min	97%	98%	56%
30min	98%	98%	80%

Reaction conditions: 0.1 mmol **1**, 1.0 mol% catalyst, H_2 (1 bar), 2 ml ^{i}PrOH, 30 min, r.t.

General procedure for the hydrogenation of ketones

An oven-dried glass vial equipped with magnetic stirring bar was charged with substrate (0.1 mmol), iridium complex (1.0 mol%) and dry ^{i}PrOH (2.0 ml). The vial was placed in a low-pressure hydrogenation apparatus, purged three times with nitrogen, purged three times with hydrogen gas and then pressurized to 1.0 bar with hydrogen gas. After stirring for 30 min at room temperature, the pressure was released and the solvent was removed under vacuum. The crude product was then purified by column chromatography on silica gel (pentane/Et_2O, 1/1) to give the corresponding product. Conversions were determined by ^{1}H NMR spectroscopy and the *ee* values were determined by GC or SFC analysis using a chiral stationary phase. The configuration of the products was determined by comparing their optical rotation with literature data.

Procedure for the hydrogenation of acetophenone 1 using pre-activated catalyst.

An oven-dried glass vial equipped with magnetic stirring bar was charged with iridium complex (1.0 mol%) and dry ^{i}PrOH (1.0 ml). The vial was sealed and purged three times with nitrogen, purged three times with hydrogen and then pressurized with a balloon of H_2. After string for 15 min, a color change from red to light yellow was observed. Acetophenone **1** (0.1 mmol) in ^{i}PrOH (1.0 ml) was injected and the reaction mixture was stirred under a balloon of H_2 and at room temperature for another 5 min. The balloon of H_2 was removed and the solvent was evaporated. The conversion was determined by ^{1}H NMR spectroscopy and the *ee* value was determined by GC using a chiral stationary phase. The configuration of the products was determined by comparing their optical rotations with literature data.

Preparation of iridium complexes

Preparation of achiral NHC-phoshphine iridium complexes Ir-A to Ir-H

Achiral NHC-phosphine iridium complexes **Ir-A** to **Ir-H** were prepared according to reported procedures.[1] Spectroscopic data of **Ir-A**,[2] **Ir-B**,[1] **Ir-C**,[1] **Ir-D**[3] and **Ir-E**[1] were in accordance to reported data.

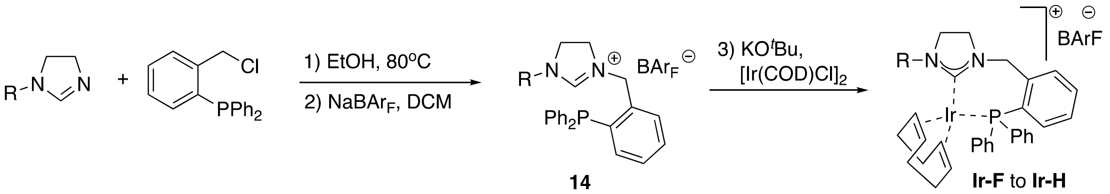

Synthesis of imidazoline derivatives 14f-h

To a Schlenktube was added the corresponding imidazoline (1.1 equiv.), followed by the triphenylphosphine derivative (1.0 equiv.) in EtOH (0.1M) under argon atmosphere. The mixture was allowed to stir at 80 °C for 12 h after which EtOH was removed under vacuum. The remaining compound was dissolved in DCM (0.1M) followed by the addition of NaBAr$_F$ (1.2 equiv.). After stirring at room temperature for 1 h, the mixture was filtrated through Celite and the solvent was removed under vacuum. The resulting residue was purified by column chromatography (DCM/pentane, 3/1) to obtain pure compounds **14f-h**.

Imidazolium salt 14f

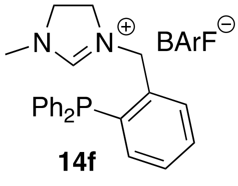

14f

Yield: 57%, colorless oil. **R$_f$** = 0.42 (DCM). **^{1}H NMR** (CDCl$_3$, 400 MHz): δ 7.77 – 7.68 (m, 8H), 7.54 (s, 4H), 7.45 – 7.32 (m, 8H), 7.30 – 7.01 (m, 7H), 4.80 (s, 2H), 3.47 (dd, *J* = 12.6, 9.4 Hz, 2H), 3.09 (dd, *J* = 12.6, 9.4 Hz, 2H), 2.56 (s, 3H). **^{13}C NMR** (CDCl$_3$, 100 MHz): δ 161.8 (dd, *J* = 99.8, 49.9 Hz), 156.3 (d, *J* = 4.3 Hz), 137.0 (d, *J* = 14.7 Hz), 136.2, 135.0 – 134.7 (m), 134.5 (d, *J* = 6.6 Hz), 133.8, 133.6, 131.4 (d, *J* = 5.2 Hz), 131.0, 130.6, 129.9, 129.6 – 129.2 (m), 129.0 – 128.8 (m), 128.7 – 128.4 (m), 126.0, 123.3, 117.7, 52.1 (d, *J* = 19.5 Hz), 49.9, 47.9, 34.7. **^{31}P NMR** (CDCl$_3$, 162 MHz): δ -19.0. **IR** (neat, cm^{-1}): ν = 3055, 2986, 1663, 1355, 1278, 1126, 895, 744. **HRMS-ESI** calcd for $C_{23}H_{24}N_2P$ [M-BAr$_F$]$^+$: 359.1672, found 359.1680.

Imidazolium salt 14g

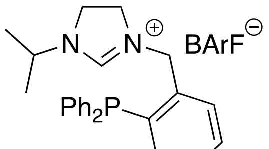

14g

Yield: 62% yield, colorless oil. **R$_f$** = 0.40 (DCM). **^{1}H NMR** ($CDCl_3$, 400 MHz): δ 7.74 (brs, 8H), 7.56 (brs, 4H), 7.46 – 7.34 (m, 8H), 7.29 – 7.14 (m, 6H), 7.11 (ddd, J = 6.9, 4.5, 1.9 Hz, 1H), 4.80 (s, 2H), 3.50 (dd, J = 12.7, 9.1 Hz, 2H), 3.33 – 3.19 (m, 3H), 1.04 (d, J = 6.6 Hz, 6H). **^{13}C NMR** ($CDCl_3$, 100 MHz): δ 161.8 (dd, J = 99.6, 49.8 Hz), 154.1 (d, J = 5.4 Hz), 138.0, 136.8 (d, J = 14.5 Hz), 136.2, 134.9 (d, J = 4.6 Hz), 134.6 (d, J = 6.4 Hz), 133.6, 133.4, 131.2 (d, J = 5.3 Hz), 130.9, 130.6, 129.9, 129.5 – 129.1 (m), 129.0 – 128.8 (m), 128.4, 126.0, 125.4, 123.3, 120.6, 117.8 – 117.2 (m), 52.3 (d, J = 19.0 Hz), 51.2, 47.4, 46.0, 21.6, 20.2. **^{31}P NMR** ($CDCl_3$, 162 MHz): δ-18.8. **IR** (neat, cm^{-1}): ν = 3056, 2987, 1648, 1355, 1278, 1125, 745. **HRMS-ESI** calcd for $C_{25}H_{28}N_2P$ [M-BAr$_F$]$^+$: 387.1985, found 387.2007.

Imidazolium salt 14h

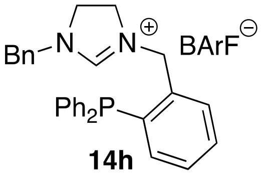

14h

Yield: 58%, colorless oil. **R$_f$** = 0.45 (DCM). **^{1}H NMR** ($CDCl_3$, 400 MHz): δ 7.72 (s, 8H), 7.52 (s, 4H), 7.47 – 7.31 (m, 12H), 7.22 – 7.14 (m, 5H), 7.07 (ddt, J = 9.6, 6.5, 1.7 Hz, 3H), 4.75 (s, 2H), 4.06 (s, 2H), 3.50 (dd, J = 12.6, 9.2 Hz, 2H), 3.24 (dd, J = 12.6, 9.2 Hz, 2H). **^{13}C NMR** ($CDCl_3$, 100 MHz: δ 161.8 (dd, J = 99.6, 50.0 Hz), 155.6 (d, J = 5.7 Hz), 136.9 (d, J = 14.7 Hz), 136.1, 135.0 – 134.6 (m), 134.4 (d, J = 6.3 Hz), 133.8, 133.6, 131.3 (d, J = 5.2 Hz), 131.0, 130.6, 130.4, 130.0, 129.9, 129.3 – 129.1 (m), 128.9 – 128.8 (m), 128.6, 126.0, 123.3, 120.6, 117.9 – 117.3 (m), 100.1, 52.9, 52.4 (d, J = 18.3 Hz), 48.1, 47.8. **^{31}P NMR** ($CDCl_3$, 162 MHz): δ -18.5. **IR** (neat, cm^{-1}): ν = 3054, 2987, 1651, 1421, 1265, 745. **HRMS-ESI** calcd for $C_{29}H_{28}N_2P$ [M-BAr$_F$]$^+$: 435.1985, found 435.1979.

Synthesis of achiral NHC-phosphine iridium complexes Ir-F to Ir-H

Compound **14** (1.0 equiv.) was dissolved in dry THF (0.05M) under argon, followed by the addition of $[Ir(COD)Cl]_2$ (0.5 equiv.) and KO^{t}Bu (1.05 equiv.) and the mixture was allowed to stir at room temperature for 3 h. The solvent was removed under vacuum and the residue was purified by flash column chromatography on silica gel (DCM/pentane, 1/1 to 3/1) to obtain catalysts **Ir-F** to **Ir-H** as red solids.

NHC-phosphine iridium complex Ir-F

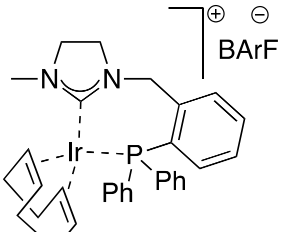

Ir-F

Yield: 76%, red solid. **R$_f$** = 0.80 (DCM). **^{1}H NMR** ($CDCl_3$, 400 MHz): δ 7.80 – 7.69 (m, 8H), 7.55 (s, 4H), 7.52 – 7.45 (m, 4H), 7.44 – 7.36 (m, 3H), 7.36 – 7.24 (m, 5H), 7.10 (dd, *J* = 11.1, 7.8 Hz, 1H), 6.76 (dd, *J* = 11.4, 3.8 Hz, 1H), 5.00 – 4.90 (m, 1H), 4.48 – 4.39 (m, 1H), 4.08 – 3.97 (m, 1H), 3.90 – 3.80 (m, 1H), 3.43 – 3.20 (m, 2H), 3.02 – 2.72 (m, 2H), 2.53 – 2.29 (m, 4H), 2.23 (s, 3H), 2.19 – 2.02 (m, 4H), 1.96 – 1.73 (m, 2H). **^{13}C NMR** ($CDCl_3$, 100 MHz): δ 198.8 (d, *J* = 10.8 Hz),□161.7 (dd, *J* = 99.7, 49.8 Hz), 144.2 (d, *J* = 12.3 Hz), 139.4 (d, *J* = 2.7 Hz), 134.8, 133.0, 132.1 – 131.4 (m), 131.1 – 130.7 (m), 129.2 – 128.1 (m), 127.7, 127.0 (d, *J* = 8.5 Hz), 125.9, 123.2, 120.5, 117.8 – 117.0 (m), 88.3 (d, *J* = 10.0 Hz), 85.3 (d, *J* = 12.9 Hz), 80.2, 78.5, 62.3 (d, *J* = 8.1 Hz), 51.5, 42.3, 35.5, 34.9 – 33.1 (m), 28.5 (dd, *J* = 36.9, 2.2 Hz), 22.4, 11.3. **^{31}P NMR** ($CDCl_3$, 162 MHz): δ 7.4. **IR** (neat, cm^{-1}): ν = 3054, 2987, 1608, 1421, 1265, 896, 740. **HRMS-ESI** calcd for $C_{31}H_{35}IrN_2P$ [M-BAr$_F$]$^+$: 659.2162, found 659.2167.

NHC-phosphine iridium complex Ir-G

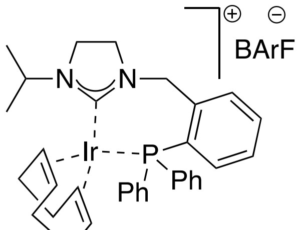

Ir-G

Yield: 70%, red solid. **R$_f$** = 0.82 (DCM). **^{1}H NMR** ($CDCl_3$, 400 MHz): δ 7.79 – 7.69 (m, 8H), 7.61 – 7.45 (m, 10H), 7.42 – 7.29 (m, 5H), 7.21 – 7.11 (m, 1H), 7.09 – 6.99 (m, 2H), 6.67 (d, *J* = 14.6 Hz, 1H), 5.02 – 4.96 (m, 1H), 4.55 – 4.44 (m, 1H), 4.16 – 3.96 (m, 3H), 3.70 – 3.61 (m, 1H), 3.47 (td, *J* = 9.8, 4.6 Hz, 1H), 3.27 (q, *J* = 10.2 Hz, 1H), 3.15 (td, *J* = 10.9, 8.9 Hz, 1H), 3.03 (ddd, *J* = 11.0, 9.2, 4.6 Hz, 1H), 2.44 (dtd, *J* = 29.9, 9.2, 5.8 Hz, 4H), 2.18 – 2.06 (m, 1H), 2.04 – 1.93 (m, 1H), 1.91 – 1.81 (m, 1H), 1.73 – 1.59 (m, 1H), 1.01 (d, *J* = 6.6 Hz, 3H), 0.94 – 0.81 (m, 1H), -0.01 (d, *J* = 6.6 Hz, 3H). **^{13}C NMR** ($CDCl_3$, 100 MHz): δ 197.9 (d, *J* = 10.6 Hz), 161.9 (dd, *J* = 99.7, 49.9 Hz), 141.6 (d, *J* = 13.5 Hz), 139.9 (d, *J* = 1.9 Hz), 134.9, 133.4 (d, *J* = 11.4 Hz), 132.4 – 131.8 (m), 131.2, 130.9 (d, *J* = 2.4 Hz), 130.7, 129.8, 129.7, 129.6 – 129.5 (m), 129.4 – 129.1 (m), 128.9 (dt, *J* = 5.7, 2.8 Hz), 128.7 (d, *J* = 4.5 Hz), 128.7 – 128.3 (m), 126.1, 123.4, 120.7, 118.3 – 116.7 (m), 89.8 (d, *J* = 9.3 Hz), 84.5 (d, *J* = 13.7 Hz), 79.8 (d, *J* = 7.9 Hz), 54.8 (d, *J* = 7.1 Hz), 50.5, 47.1, 43.0, 35.0 (d, *J* = 1.8 Hz), 34.4 (d, *J* = 4.0 Hz), 28.3 (d, *J* = 2.2 Hz), 27.7 (d, *J* = 2.1 Hz), 21.7, 19.1. **^{31}P NMR** ($CDCl_3$, 162 MHz): δ 6.1. **IR** (neat,

cm^{-1}): ν = 2985, 1610, 1495, 1354, 1277, 1125, 748. **HRMS-ESI** calcd for $C_{33}H_{39}IrN_2P$ [M-BAr$_F$]$^+$: 687.2476, found 687.2474.

NHC-phosphine iridium complex Ir-H

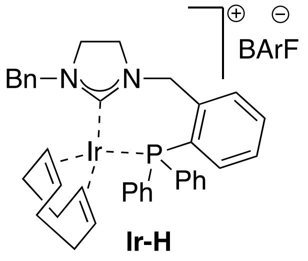

Ir-H

Yield: 72%, red solid. **R$_f$** = 0.80 (DCM). **^{1}H NMR** ($CDCl_3$, 400 MHz): *δ* 7.79 – 7.68 (m, 9H), 7.61 – 7.55 (m, 3H), 7.55 – 7.49 (m, 5H), 7.49 – 7.31 (m, 5H), 7.30 – 7.19 (m, 5H), 7.19 – 7.09 (m, 1H), 6.84 – 6.77 (m, 2H), 6.69 – 6.54 (m, 1H), 5.14 – 5.09 (m, 1H), 4.96 (dd, *J* = 15.8, 9.4 Hz, 1H), 4.45 (q, *J* = 6.8 Hz, 1H), 4.15 (dd, *J* = 14.8, 8.8 Hz, 1H), 4.06 – 3.85 (m, 2H), 3.64 – 3.41 (m, 1H), 3.40 – 3.15 (m, 2H), 2.73 – 2.54 (m, 2H), 2.52 – 2.26 (m, 4H), 2.20 – 1.92 (m, 1H), 1.90 – 1.68 (m, 1H), 1.40 – 1.16 (m, 3H). **^{13}C NMR** ($CDCl_3$, 100 MHz): *δ* 200.3 (d, *J* = 9.8 Hz), 161.9 (dd, *J* = 99.9, 50.1 Hz), 141.8 (d, *J* = 12.8 Hz), 139.4 (d, *J* = 2.0 Hz), 134.9, 134.3, 133.4 (d, *J* = 11.4 Hz), 132.5 – 132.3 (m), 132.2 – 131.9 (m), 131.7 (d, *J* = 9.0 Hz), 131.6 – 131.4 (m), 131.4 – 131.1 (m), 129.7 – 129.1 (m), 129.1 – 128.5 (m), 126.1, 123.3, 120.6, 117.6, 89.1 (d, *J* = 9.6 Hz), 85.8 (d, *J* = 13.2 Hz), 81.2, 80.1, 54.8 (d, *J* = 6.5 Hz), 53.1, 49.4, 47.6, 34.8, 34.1 (d, *J* = 3.9 Hz), 29.9, 28.6 (d, *J* = 2.1 Hz), 28.2 (d, *J* = 2.2 Hz). **^{31}P NMR** ($CDCl_3$, 162 MHz): *δ* 7.5. **IR** (neat, cm^{-1}): ν = 2925, 1609, 1354, 1277, 1125, 748. **HRMS-ESI** calcd for $C_{37}H_{39}IrN_2P$ [M-BAr$_F$]$^+$: 735.2477, found 735.2479.

Preparation of chiral NHC-phosphine iridium complexes Ir-1 to Ir-11.

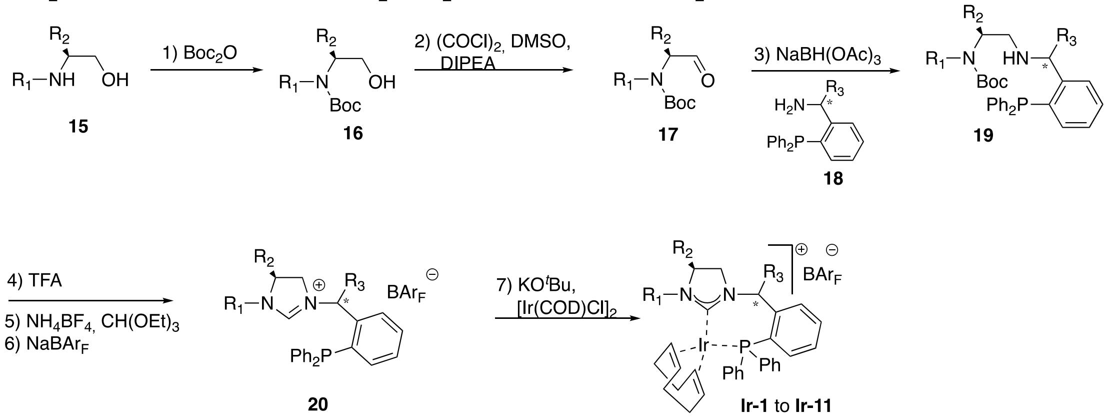

Synthesis of amino alcohols 15a-e

Amino alcohols **15a-c** are commercially available and the chiral amino alcohol **15d**[4] and **15e**[5] were prepared according to reported procedures.

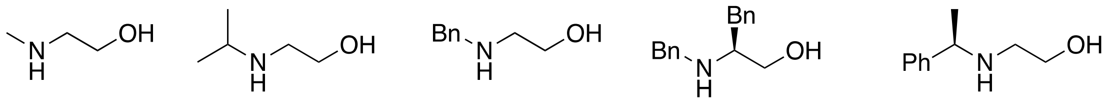

15a **15b** **15c** **15d** **15e**

Boc-protection of amino alcohols 15a-e

Boc-protected amino alcohols **16a-e** were prepared according to reported procedures.[6] Spectroscopic data of compound **16a**,[7] **16b**,[8] **16c**,[9] and **16d**[6] were in accordance with reported data.

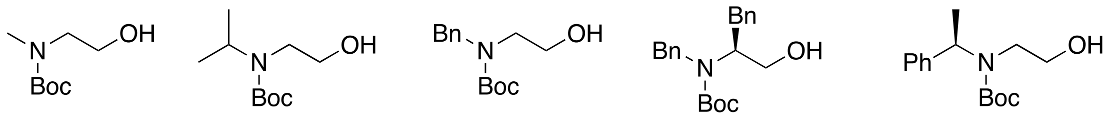

16a **16b** **16c** **16d** **16e**

t-Butyl (*R*)-(2-hydroxyethyl)(1-phenylethyl)carbamate 16e

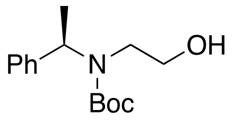

16e

Yield: 79%, colorless oil. $\mathbf{R_f}$ = 0.60 (EtOAc/Pentane, 1/3). **^{1}H NMR** ($CDCl_3$, 400 MHz): δ 7.38 – 7.23 (m, 5H), 5.35 (brs, 1H), 3.55 – 3.45 (m, 2H), 3.30 – 3.10 (m, 2H), 2.37 (brs, 1H), 1.53 (d, *J* = 7.1 Hz, 3H), 1.48 (s, 9H). **^{13}C NMR** ($CDCl_3$, 100 MHz): δ 141.6, 128.6, 127.49, 127.1, 80.8, 28.6, 17.6. **IR** (neat, cm^{-1}): ν = 3434, 2976, 2943, 1688, 1666, 1452, 1405, 1366, 1164, 1051, 699. $[\alpha]_D^{21}$ = +95.4 (*c* = 1.0, $CHCl_3$). **HRMS-ESI** calcd for $C_{15}H_{23}NNaO_3$ $[M+Na]^+$: 288.1570, found: 288.1578.

Oxidation of amino alcohols 16a-e

To a solution of oxalyl chloride (4.0 mmol) in DCM (20 ml) was added dimethylsulfoxide (8.1 mmol) at -78 °C. After stirring for 5 min at -78 °C, the reaction mixture was allowed to warm to -60 °C over 30 min followed by the slow addition of the corresponding alcohol (2.7 mmol) in DCM (10 ml) over 5 min. The reaction mixture was warmed to -45 °C over 30 min, stirred for 5 min at this temperatures and diisopropylethylamine (16.2 mmol) was added. After 5 min, the cooling bath was removed and the solution was warmed to 0 °C, then poured into a mixture of HCl (10 ml, aq. 1.0 M) and ice. The combined organic extract was washed with brine and dried with Na_2SO_4. The solvent was removed under vacuum to obtained a yellow oil which was purified by column chromatography on silica gel (EtOAc/pentane, 1/9) to

obtain the product. Spectroscopic data of compound **17a**,[10] **17b**,[10] **17c**[11] and **17d**[6] were in accordance with reported data.

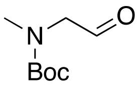

17a

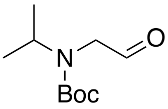

17b

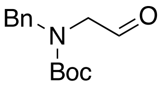

17c

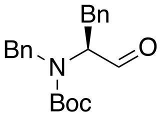

17d

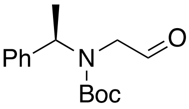

17e

***t*-Butyl (*R*)-(2-oxoethyl)(1-phenylethyl)carbamate 17e**

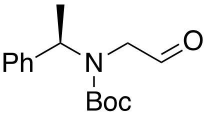

17e

Yield: 97%, colorless oil. **R$_f$** = 0.56 (EtOAc/Pentane, 1/9). **^{1}H NMR** ($CDCl_3$, 400 MHz): δ 9.31 (s, 1H), 7.68 – 6.85 (m, 5H), 5.96 – 5.35 (m, 1H), 4.25 – 3.11 (m, 2H), 1.52 (d, *J* = 7.1 Hz, 3H), 1.49 (s, 9H). **^{13}C NMR** ($CDCl_3$, 100 MHz): δ 199.7, 128.8, 127.9, 127.4, 81.2, 52.3, 28.4, 16.8. **IR** (neat, cm^{-1}): ν = 3013, 2979, 2816, 2709, 1736, 1691, 1430, 1392, 1377, 1166, 860, 756. $[\alpha]_D^{21}$ = +88.0 (*c* = 1.0, $CHCl_3$). **HRMS-EI** calcd for $C_{15}H_{21}NNaO_3$ $[M+Na]^+$: 286.1414, found: 286.1400.

Synthesis of the chiral amine phosphine 18a-d.

Amine phosphines **18a-d** were prepared according to reported procedures.[12] Spectroscopic data of compound **18a** and **18b** were in accordance with reported data.[12]

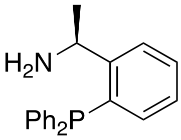

18a

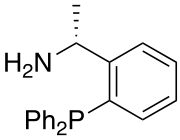

18b

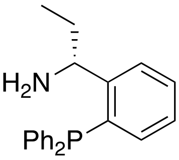

18c

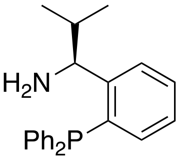

18d

(*R*)-1-(2-(Diphenylphosphanyl)phenyl)propan-1-amine 18c

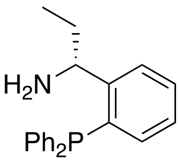

18c

Yield: 26%, pink oil. **R$_f$** = 0.20 (DCM/Pentane/MeOH, 1/1/0.01). **^{1}H NMR** ($CDCl_3$, 400 MHz): δ 7.53 (m, 1H), 7.40-7.21 (m, 11H), 7.13 (m, 1H), 6.88 (m, 1H), 4.67 (m, 1H), 2.10 (brs, 2H), 1.63 (m, 2H), 0.77 (t, *J* = 7.6 Hz, 3H). **^{13}C NMR** ($CDCl_3$, 100 MHz): δ 137.0 (dd, *J* = 51.0, 10 Hz), 135.1 (d, *J* =12 Hz), 134.0 (dd, *J* = 41 Hz, 20 Hz), 133.5, 129.5, 128.7 (d, *J* = 12 Hz), 128.6, 128.5, 127.1, 125.9, 54.2, 31.1, 10.9. **^{31}P NMR** ($CDCl_3$, 162 MHz): δ -16.5. **IR** (neat, cm^{-1}): ν = 2977, 1807, 1736, 1695, 1367,

1166, 700, 486. $[\alpha]_D^{21}$ = +42.4 (c = 0.5, $CHCl_3$). **HRMS-ESI** calcd for $C_{21}H_{23}NP$ $[M+H]^+$: 320.1563, found 320.1563.

(*S*)-1-(2-(Diphenylphosphanyl)phenyl)-2-methylpropan-1-amine 18d

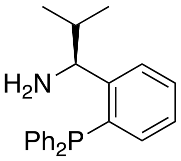

18d

Yield: 38%, yellow oil. This compound was obtained after column chromatography with some impurities and further purification could not give the pure compound. The compound was directly used in the next step. R_f = 0.15 (DCM/Pentane/MeOH, 1/1/0.01). **^{1}H NMR** ($CDCl_3$, 400 MHz): δ 7.53 (m, 1H), 7.40-7.21 (m, 11H), 7.10 (m, 1H), 6.90 (m, 1H), 4.50 (m, 1H), 1.98 (m, 1H), 1.05 (d, J = 7.1 Hz, 3H), 0.71 (d, J = 7.1 Hz, 3H). **^{31}P NMR** ($CDCl_3$, 162 M Hz): δ -16.7. **HRMS-ESI** calcd for $C_{22}H_{25}NP$ $[M+H]^+$: 334.1791, found 334.1736.

Reductive amination of 17 with 18

Boc-protected diamine phosphines **19a-k** were prepared according to reported procedures.[12] A solution of aldehyde (1.3 mmol) in 1,2-dichloroethane (5 ml) was added to a solution of the corresponding amine (1.0 mmol) and sodium triacetoxyborohydride (2.0 mmol) in 1,2-dichloroethane (5 ml). The mixture was stirred at room temperature for 4 h and quenched with 10% $NaHCO_3$ (10 ml). The aqueous phase was separated and extracted with DCM (3×10 ml) and the combined organic extract was washed with brine and dried with Na_2SO_4. The solvent was removed under vacuum to obtain the crude, which was purified by column chromatography on silica gel (EtOAc/pentane, 1/10) to obtain the product.

Boc-protected diamine phosphine 19a

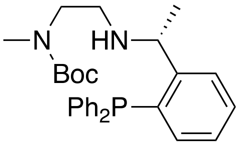

19a

Yield: 73%, light yellow oil. R_f = 0.23 (Pentane/EtOAc, 1/1). **^{1}H NMR** ($CDCl_3$, 400 MHz): δ 7.58 (ddd, J = 7.9, 4.3, 1.3 Hz, 1H), 7.37 – 7.23 (m, 11H), 7.12 (td, J = 7.5, 1.4 Hz, 1H), 6.86 (ddd, J = 7.8, 4.3, 1.4 Hz, 1H), 4.63 (p, J = 6.6 Hz, 1H), 3.22 – 3.18 (m, 1H), 3.01 (br. s, 1H), 2.75 (s, 3H), 2.47 – 2.42 (m, 2H), 1.43 (s, 9H), 1.19 (d, 3H, J = 6.4 Hz). **^{13}C NMR** ($CDCl_3$, 100 MHz): δ 155.9, 150.0 (d, J = 22.2 Hz), 137.0 (d, J = 10.5 Hz), 136.6 (d, J = 10.5 Hz), 135.1 (d, J = 13.2 Hz), 134.1, 134.00, 133.95, 133.8, 133.3, 129.4, 128.7, 128.5, 128.4, 126.9, 125.8 (d, J = 5.1 Hz) 79.2, 54.4 (d, J = 19.1

Hz) 48.9, 34.6, 28.4, 23.8. **^{31}P NMR** (CDCl$_3$, 162 MHz): δ -17.1. **IR** (neat, cm^{-1}): ν = 3322, 2974, 1693, 1479, 1434, 1392, 1158, 744. $[\alpha]_D^{21}$ = +59.0 (c = 1.0, CHCl$_3$). **HRMS-ESI** calcd for $C_{28}H_{35}N_2O_2P$ [M-BAr$_F$]$^+$: 463.2509, found 463.2498.

Boc-protected diamine phosphine 19b

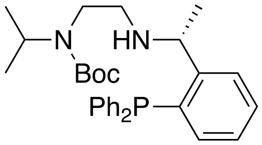

19b

Yield: 73%, white solid. **R$_f$** = 0.33 (Pentane/EtOAc, 1/1). **^{1}H NMR** (CDCl$_3$, 400 MHz): δ 7.59 – 7.68 (m, 1H), 7.24 – 7.42 (m, 11H), 7.13 (td, J = 7.5, 1.4 Hz, 1H), 6.68 (ddd, J = 7.7, 4.3, 1.4, 1H), 4.66 (m, 1H), 4.17 (brs, 1H), 3.02 (s, 2H), 2.46 (t, J= 7.1 Hz, 2H), 1.40 (s, 9H), 1.21 (d, 3H, J = 6.4 Hz), 1.02 (d, 3H, J = 8.0 Hz), 1.00 (d, 3H, J= 8.0 Hz). **^{13}C NMR** (CDCl$_3$, 100 MHz): δ 155.4, 150.0 (d, J = 22.7 Hz), 137.0, (d, J =10.6 Hz), 136.6 (d, J =10.4 Hz), 134.9 (d, J = 13.2 Hz), 134.1, 133.91, 133.89, 133.7, 133.3, 129.3, 128.63, 128.61, 128.45, 128.4, 126.9, 125.8 (d, J = 4.7 Hz), 79.0, 54.3 (d, J = 25.3 Hz), 47.2, 28.4, 23.6, 20.7. **^{31}P NMR** (162 MHz, CDCl3): δ –17.2. **IR** (neat, cm^{-1}): ν = 3327, 2973, 1687, 1585, 1477, 1408, 1365, 1167. $[\alpha]_D^{21}$ = +41.2 (c = 1.0, CHCl$_3$). **HRMS-ESI** calcd for $C_{30}H_{40}N_2O_2P$ [M+H]$^+$: 491.2822, found 491.2829.

Boc-protected diamine phosphine 19c

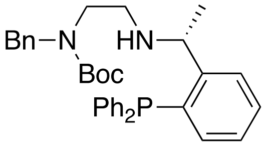

19c

Yield: 91%, colorless oil. **R$_f$** = 0.40 (Pentane/EtOAc, 5/1). **^{1}H NMR** (CDCl$_3$, 400 MHz): δ 7.51 (m, 1H), 7.39-7.13 (m, 17H), 6.86 (m, 1H), 4.62-4.28 (brm, 3H), 3.20-3.05 (m, 2H), 2.46 (m, 2H), 1.45 (m, 9H), 1.16 (d, J = 6.0 Hz, 3H). **^{13}C NMR** (CDCl$_3$, 100 MHz): δ 156.0, 138.4, 136.8 (dd, J = 36, 10 Hz), 135.2, 134.1, 134.0, 133.9, 133.8, 133.3, 129.5, 128.7, 128.5,128.4, 127.7, 127.1, 127.0, 126.0, 79.8, 54.2, 50.7 (m), 46.6, 45.4, 28.4, 23.5. **^{31}P NMR** (CDCl$_3$, 162 MHz): δ -17.1. **IR** (neat, cm^{-1}): ν = 2974, 1693, 1453, 1170, 743, 698. $[\alpha]_D^{21}$ = +27.1 (c = 1.0, CHCl$_3$). **HRMS-ESI** calcd for $C_{34}H_{40}N_2O_2P$ [M+H]$^+$: 539.2822, Found 539.2828.

Boc-protected diamine phosphine 19d

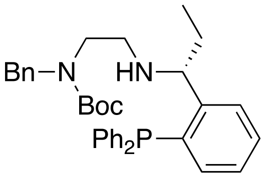

19d

Yield: 88%, colorless oil. $\mathbf{R_f}$ = 0.40 (Pentane/EtOAc, 5/1). **^{1}H NMR** ($CDCl_3$, 400 MHz): δ 7.52 (m, 2H), 7.40-7.14 (m, 16H), 6.94 (m, 1H), 4.51-4.30 (m, 3H), 3.21-3.03 (m, 2H), 2.60-2.43 (m, 2H), 1.64 (m, 2H), 1.47 (s, 9H), 0.76 (t, *J* = 7.6 Hz, 3H). **^{13}C NMR** ($CDCl_3$, 100 MHz): δ 156.0, 149.2, 138.4, 137.0 (dd, *J* = 37, 11 Hz), 135.8 (d, *J* = 13 Hz), 134.1, 133.9, 133.7, 133.6, 132.2 (m), 131.6, 129.2, 128.6, 128.3, 128.2, 127.6-127.0 (m), 79.5, 60.4, 50.4, 46.7, 45.4, 30.7, 28.3, 10.8. **^{31}P NMR** ($CDCl_3$, 162 M Hz): δ -17.6. **IR** (neat, cm^{-1}): ν = 2972, 1690, 1414, 1366, 1170, 740, 698. $[\alpha]_D^{21}$ = +26.5 (*c* = 1.0, $CHCl_3$). **HRMS-ESI** calcd for $C_{35}H_{42}N_2O_2P$ $[M+H]^+$: 553.2978, found 553.2958.

Boc-protected diamine phosphine 19e

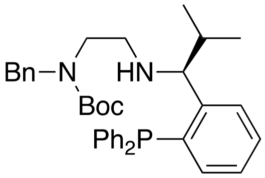

19e

Yield: 70%, colorless oil. $\mathbf{R_f}$ = 0.40 (Pentane/EtOAc, 5/1). **^{1}H NMR** ($CDCl_3$, 400 MHz): δ 7.52 (m, 1H), 7.40-7.14 (m, 16H), 7.01 (m, 1H), 4.50-4.30 (m, 3H), 3.40-3.01 (m, 2H), 2.54-2.21 (m, 2H), 1.98 (m, 1H), 1.47 (m, 9H), 1.02 (d, *J* = 6.8 Hz, 3H), 0.77 (d, *J* = 6.8 Hz, 3H). **^{13}C NMR** ($CDCl_3$, 100 MHz): δ 156.0, 138.4, 137.0 (dd, *J* = 37, 11 Hz), 136.1 (d, *J* = 13 Hz), 134.2, 133.9, 133.8, 133.7, 133.6, 129.1, 128.4-128.3 (m), 127.9, 127.2-126.8 (m), 126.5, 79.4, 65.2, 50.1, 46.6, 45.6, 34.5, 28.3, 20.3, 14.1. **^{31}P NMR** ($CDCl_3$, 162 MHz): δ -18.2. **IR** (neat, cm^{-1}): ν = 2971, 1693, 1413, 1169, 742, 698. $[\alpha]_D^{21}$ = −33.8 (*c* = 1.0, $CHCl_3$). **HRMS-ESI** calcd for $C_{36}H_{44}N_2O_2P$ $[M+H]^+$: 567.3135, found 567.3126.

Boc-protected diamine phosphine 19f

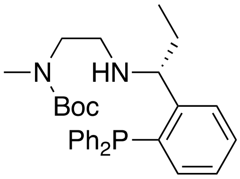

19f

Yield: 74 %, white solid. $\mathbf{R_f}$ = 0.33 (Pentane/EtOAc, 1/1). **^{1}H NMR** ($CDCl_3$, 400 MHz): δ 7.51 (dd, *J* = 8.1, 4.2 Hz, 1H), 7.22 – 7.38 (m, 11H), 7.13 (td, *J* = 7.5, 1.4 Hz, 1H), 6.91 (ddd, *J* = 7.8, 4.2, 1.4 Hz, 1H), 4.48 (q, *J* = 6.8 Hz, 1H), 3.25 – 3.13 (m, 1H), 3.00 (br. s, 1H), 2.72 (s, 3H), 2.46 – 2.31 (m, 2H), 1.68 – 1.53 (m, 2H), 1.42 (s, 9H) 0.74 (t, *J* = 7.4 Hz, 3H). **^{13}C NMR** ($CDCl_3$, 100 MHz): δ 155.8, 149.1, 137.1 (d, *J* = 11.0 Hz), 136.8 (d, *J* = 10.8 Hz), 137.0, (d, *J* =13.1 Hz), 134.1, 133.89, 133.87, 133.7, 133.6, 129.2, 128.5, 128.4, 128.3, 126.7, 126.1-126.5 (m), 79.1, 60.5 (d, *J* =

23.5 Hz), 48.9, 45.2, 34.5, 30.7, 28.4, 10.7. **^{31}P NMR** (162 MHz, CDCl3): δ –17.6. **IR** (neat, cm^{-1}): ν = 3338, 3053, 2970, 1693, 1479, 1393, 1158, 744, 698. $[\alpha]_D^{21}$ = +67.6 (*c* = 1.0, CHCl3). **HRMS-ESI** calcd for $C_{29}H_{38}N_2O_2P$ $[M+H]^+$ 477.2665, found 477.2688.

Boc-protected diamine phosphine 19g

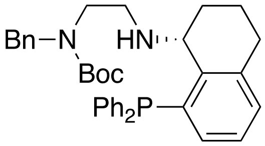

19g

Yield: 60%. This compound was obtained after column chromatography with some impurities and further purification could not give the pure compound. The compound was directly used for the next step. **^{1}H NMR** ($CDCl_3$, 400 MHz): δ 7.62 (m, 1H), 7.54-6.97 (m, 17H), 6.88 (m, 1H), 4.63 (m, 1H), 4.17 (m, 2H), 3.48-3.20 (m, 1H), 2.98 (m, 1H), 2.70-2.43 (m, 4H), 1.80-1.71 (m, 2H), 1.48-1.41 (m, 11H). **^{31}P NMR** ($CDCl_3$, 162 MHz): δ -17.5.

Boc-protected diamine phosphine 19h

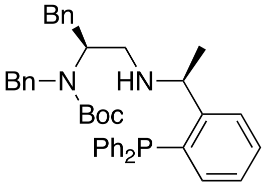

19h

Yield: 91%, light yellow oil. **R$_f$** = 0.80 (Pentane/EtOAc, 1/1). **^{1}H NMR** ($CDCl_3$, 400 MHz): δ 7.58 (m, 1H) 7.39-7.11 (m, 22H), 6.88 (m, 1H), 4.32-4.17 (m, 4H), 2.78 (m, 2H), 2.42 (m, 2H), 1.44 (m, 9H), 1.02 (m, 3H). **^{13}C NMR** ($CDCl_3$, 100 MHz): δ 156.1 (m), 150.1, 139.6, 138.8, 136.8 (dd, *J* = 51, 11 Hz), 134.7 (d, *J* = 14 Hz), 134.1, 133.9, 133.7, 133.0, 129.2, 129.0, 128.5, 128.4, 128.3 (m), 128.2, 128.1, 127.8, 127.1, 126.9, 126.9, 126.6, 126.0, 125.9, 79.5, 59.0 (m), 54.8 (d, *J* = 24 Hz), 49.5, 48.5, 37.7, 28.2, 23.6. **^{31}PNMR** ($CDCl_3$, 100 MHz): δ -16.9. **IR** (neat, cm^{-1}): ν = 2926, 1642, 1355, 1278, 1127, 744, 682. $[\alpha]_D^{21}$ = +86.0 (*c* = 1.0, CHCl3). **HRMS-ESI** calcd for $C_{41}H_{46}N_2O_2P$ $[M+H]^+$: 629.3291, found 629.3270.

Boc-protected diamine phosphine 19i

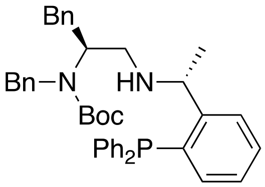

19i

Yield: 94%, light yellow oil. **R$_f$** = 0.80 (Pentane/EtOAc, 1/1). **^{1}H NMR** ($CDCl_3$, 400 MHz): δ 7.35-7.22 (m, 8H), 7.20-7.14 (m, 14H), 7.12 (m, 1H), 6.87 (m, 1H), 4.60-4.29 (m, 2H), 4.10-3.83 (m, 2H), 2.89-2.36 (m, 4H), 1.44 (m, 9H), 0.98 (d, *J* = 6.0 Hz, 3H). **^{13}C NMR** ($CDCl_3$, 100 MHz): δ 156.4, 150, 139.5, 139.0, 136.9 (dd, *J* =35, 11 Hz), 134.8 (d, *J* = 14 Hz), 134.9, 134.8, 133.9, 133.8,

133.7, 133.2, 129.4, 129.1, 128.6, 128.5, 128.4 (m), 128.2, 127.9, 127.3, 126.8, 126.0, 125.9, 79.9, 59.8, 53.6, 49.0, 38.3, 28.4, 23.7, 14.2. **^{31}P NMR** ($CDCl_3$, 162 MHz): δ -17.3. **IR** (neat, cm^{-1}): ν = 2973, 1688, 1434, 1365, 1166, 743, 698. $[\alpha]_D^{21}$ = +30.8 (*c* = 1.0, $CHCl_3$). **HRMS-EI** calcd for $C_{41}H_{46}N_2O_2P$ $[M+H]^+$: 629.3291, found: 629.3266.

Boc-protected diamine phosphine 19j

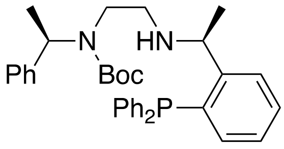

19j

Yield: 40%, colorless oil. **R$_f$** = 0.50 (EtOAc/Pentane, 1/3). **^{1}H NMR** ($CDCl_3$, 400 MHz): δ 7.48 – 7.41 (m, 1H), 7.36 – 7.17 (m, 16H), 7.11 (td, *J* = 7.5, 1.2 Hz, 1H), 6.82 (ddd, *J* = 7.7, 4.3, 1.2 Hz, 1H), 5.43 (s, 1H), 4.56 – 4.43 (m, 1H), 3.00 (s, 1H), 2.84 (s, 1H), 2.40 – 2.23 (m, 2H), 1.42-1.39 (m, 12H), 1.07 (d, *J* = 6.3 Hz, 3H). **^{13}C NMR** ($CDCl_3$, 100 MHz): δ 155.7, 150.0 (d, *J* = 22.1 Hz), 141.8, 137.0 (d, *J* = 10.8 Hz), 136.6 (d, *J* = 10.6 Hz), 134.8 (d, *J* = 13.2 Hz), 134.0, 133.9, 133.8, 133.6, 133.2, 129.3, 128.6, 128.40, 128.38, 128.33, 128.31, 128.1, 126.9, 126.7, 125.6 (d, *J* = 4.6 Hz), 79.4, 54.01 (d, *J* = 25.2 Hz), 52.6 (brs), 46.7, 43.4 (brs), 28.3, 23.5, 17.3. **^{31}P NMR** (162 MHz, CDCl3): δ 17.0. **IR** (neat, cm^{-1}): ν = 3330, 2974, 1685, 1405, 1140, 744. $[\alpha]_D^{21}$ = -7.6 (*c* = 0.5, $CHCl_3$). **HRMS-ESI** calcd for $C_{35}H_{42}N_2O_2P$ $[M+H]^+$: 553.2978, Found 553.2971.

Boc-protected diamine phosphine 19k

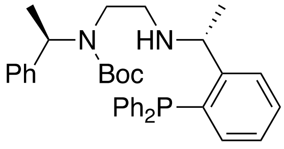

19k

Yield: 50%, colorless oil. **R$_f$** = 0.47 (EtOAc/Pentane, 1/3). **^{1}H NMR** ($CDCl_3$, 400 MHz): δ 7.77 – 7.67 (m, 8H), 7.58 – 7.51 (m, 4H), 7.51 – 7.30 (m, 12H), 7.25 – 7.17 (m, 4H), 7.15 – 7.04 (m, 3H), 5.73 (p, *J* = 6.9 Hz, 1H), 4.23 (q, *J* = 6.9 Hz, 1H), 3.68 – 3.54 (m, 1H), 3.45 – 3.30 (m, 2H), 3.28 – 3.18 (m, 1H), 1.61 (d, *J* = 6.9 Hz, 3H), 1.50 (d, *J* = 6.9 Hz, 3H). **^{13}C NMR** ($CDCl_3$, 100 MHz): δ 161.6 (dd, *J* = 99.6, 49.9 Hz), 153.4 (d, *J* = 5.6 Hz), 139.1, 138.8, 135.9, 135.4, 134.9 – 134.7 (m), 133.7, 133.5 (d, *J* = 2.8 Hz), 133.3 , 130.0 – 129.6 (m), 129.2 (dd, *J* = 14.5, 7.4 Hz), 129.1 – 128.8 (m), 128.8 – 128.6 (m), 126.7 – 126.0 (m), 125.9, 123.2, 120.5, 118.4 – 116.9 (m), 59.0, 55.7 (d, *J* = 24.3 Hz), 47.0, 45.3, 19.3, 18.0. **^{31}P NMR** ($CDCl_3$, 162 MHz): δ -17.1. **IR** (neat, cm^{-1}): ν = 3055, 2975, 2930, 1682, 1453, 1406, 1392, 1366, 1172, 1149, 909, 733, 698. $[\alpha]_D^{21}$ = +50.4 (*c* = 0.5, $CHCl_3$). **HRMS-EI** calcd for $C_{35}H_{42}N_2O_2P$ $[M+H]^+$: 553.2978, Found 553.3002.

Preparation of the imidazolium salts 20a-k

TFA (25 mmol) was added to a solution of starting material **19** (0.5 mmol) in DCM (10 ml) at 0 °C. The reaction mixture was stirred at room temperature for 20 h and then diluted with water (20 ml). The mixture was basified by sodium hydroxide solution (5M) until pH = 10. The aqueous layer was separated and extracted with DCM (2×15 ml). The combined organic extract was washed with brine, dried with $MgSO_4$ and the solvent was removed under vacuum to obtain the crude as a yellow oil which was used directly to the next step.

A mixture of previous obtained yellow oil (1.0 equiv.), triethylorthoformate (40.0 equiv.) and NH_4BF_4 (1.05 equiv.) in a pre-dried Schlenk flask was heated to 110 °C for 1 h under the argon atmosphere. After cooling to the room temperature, the precipitate was decanted and dissolved in DCM. $NaBAr_F$ (1.1 equiv.) was added and the reaction mixture was stirred at room temperature for another 30 min. After the reaction was completed, the solvent was removed under vacuum and the residue was purified by flash column chromatography on silica gel (DCM/pentane, 1/1) to obtain the product.

Imidazolium salt 20a

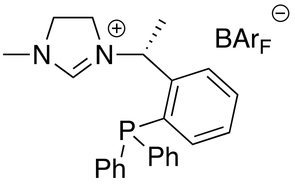

20a

Yield: 45%, colorless oil; **R$_f$** = 0.40 (DCM). **^{1}H NMR** ($CDCl_3$, 400 MHz): δ 7.69 – 7.68 (m, 8H), 7.53 (s, 4H), 7.46 – 7.34 (m, 9H), 7.22 – 7.18 (m, 2H), 7.14 – 7.07 (m, 2H), 6.91 (s, 1H), 5.74 – 5.67 (m, 1H), 3.58 (q, *J* = 11.5 Hz), 3.36 (q, *J* = 12.0 Hz, 1H), 3.17 – 3.03 (m, 1H), 2.52 (s, 3H), 1.63 (d, 3H, *J* = 6.9 Hz). **^{13}C NMR** ($CDCl_3$, 100 MHz): δ 161.6 (q, *J* = 49.5 Hz), 155.2 (d, *J* = 3.2 Hz), 139.2, 138.9, 136.6 (d, *J* = 14.8 Hz), 136.0, 134.7 – 134.6 (m), 134.1 (d, *J* = 6.3 Hz), 134.0, 133.8, 133.5, 133.3, 130.5, 130.3, 129.9, 129.7, 129.4, 129.3, 129.1 – 129.0 (m), 128.8 – 128.7 (m), 128.6, 128.5 – 128.4 (m), 126.4 (d, *J* = 4.8 Hz), 125.8, 123.1, 120.4, 117.59 – 117.47 (m), 55.6 (d, *J* = 25.4 Hz), 49.6, 45.9, 34.5, 17.9. **^{31}P NMR** ($CDCl_3$, 162 MHz): δ 8.29. **IR** (neat, cm^{-1}) ν = 1659, 1355, 1278, 1124. $[\alpha]_D^{21}$ = -12.1 (*c* =1.0, $CHCl_3$). **HRMS-ESI** calcd for $C_{24}H_{26}N_2P$ [M-BAr_F]$^+$: 373.1828, found 373.1831.

Imidazolium salt 20b

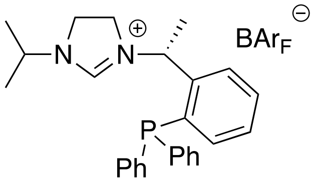

20b

Yield: 36%, light yellow oil. **R_f** = 0.5 (DCM). **^{1}H NMR** ($CDCl_3$, 400 MHz): δ 7.78 – 7.66 (m, 8H), 7.58 – 7.52 (m, 4H), 7.40 – 7.32 (m, 9H), 7.28 (s, 1H), 7.22 – 7.13 (m, 4H), 7.10 – 7.07 (m, 1H), 5.72 (p, J = 7.1 Hz, 1H), 3.67 – 3.58 (m, 1H), 1.05 – 1.01 (m, 6H). **^{13}C NMR** ($CDCl_3$, 100 MHz): δ 161.6 (dd, J = 99.7, 49.8 Hz), 153.0 (d, J = 4.1 Hz) 139.4, 139.2, 135.9, 134.8, 133.8, 133.6, 133.5, 133.3130.6, 130.3, 130.0, 129.8, 129.0 – 129.4 (m), 128.7 – 128.0 (m), 128.6, 128.4 – 128.5 (m), 126.2 (d, J = 4.9 Hz), 125.9, 123.2, 120.4, 117.46 – 114.54 (m), 55.7 (d, J = 25.1 Hz), 51.1, 45.6, 45.4, 20.1, 18.1. **^{31}P NMR** ($CDCl_3$, 162 MHz): δ -19.5. **IR** (neat, cm^{-1}): ν = 3071, 2988, 1643, 1355, 1278, 1126. $[\boldsymbol{\alpha}]_{\mathbf{D}}^{\mathbf{21}}$ = – 15.9 (c =1, $CHCl_3$). **HRMS-ESI** calcd for $[M\text{-}BAr_F]^+$: 401.2141, found 401.2150.

Imidazolium salt 20c

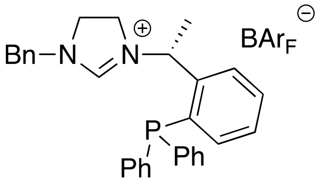

20c

Yield: 42% (in three steps), colorless oil. **R_f** = 0.25 (DCM/Pentane, 3/1). **^{1}H NMR** ($CDCl_3$, 400 MHz): δ 7.72 (brs, 8H), 7.52 (brs, 4H), 7.38-7.34 (m, 13H), 7.27 (m, 2H), 7.20 (m, 2H), 7.07 (m, 3H), 5.71 (m, 1H), 4.15 (s, 2H), 3.69 (m, 1H), 3.48-3.29 (m, 3H), 1.65 (d, J = 6.8 Hz, 3H). **^{13}C NMR** ($CDCl_3$, 100 MHz): δ 161.6 (dd, J = 99, 49 Hz), 154.60 (d, J = 4 Hz), 139.0 (d, J = 23 Hz), 136.5 (d, J = 15 Hz), 135.8, 134.9, 134.6, 134.1, 133.8 (d, J = 20 Hz), 133.4 (d, J = 20 Hz). 130.4, 130.2, 130.1, 130.0, 129.9, 129.8, 129.7, 129.3,129.2, 129.1, 129.0, 128.7 (m), 128.6, 128.4 (m), 128.3, 126.2, 125.9, 123.2, 120.5, 117.5 (m), 55.9 (d, J = 25 Hz), 52.7, 47.7, 45.9, 18.1. **^{31}P NMR** ($CDCl_3$, 162 MHz): δ-19.3. **IR** (neat, cm^{-1}): ν = 2926, 1647, 1355, 1278, 1125, 887, 682. $[\boldsymbol{\alpha}]_{\mathbf{D}}^{\mathbf{21}}$ = -11.8 (c = 0.5, $CHCl_3$). **HRMS-ESI** calcd for $C_{30}H_{43}N_2P$ $[M\text{-}BAr_F]^+$: 749.2633, found 749.2617.

Imidazolium salt 20d

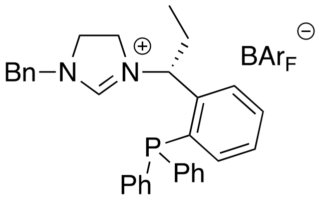

20d

Yield: 38% (in three steps), colorless oil. **R_f** = 0.25 (DCM/Pentane, 3/1). **^{1}H NMR** ($CDCl_3$, 400 MHz): δ 7.73

(brs, 8H), 7.53 (brs, 4H), 7.42-7.35 (m, 13H), 7.22-7.16 (m, 4H), 7.10-7.04 (m, 3H), 5.45 (m, 1H), 4.13 (q, J = 30.0, 14.4 Hz, 2H), 3.58 (m, 1H), 3.40 (m, 2H), 3.23 (m, 1H), 2.05 (m, 2H), 0.81 (t, J = 7.2 Hz, 3H). **^{13}C NMR** ($CDCl_3$, 100 MHz): 161.7 (dd, J = 99, 49 Hz), 154.8 (d, J = 4 Hz), 137.3 (d, J = 23 Hz), 137.0 (d, J = 15 Hz), 135.9, 134.8, 133.9, 133.74, 133.65, 133.46, 130.4, 130.3, 130.2, 130.0, 129.8 (m), 129.3, 129.2, 129.1, 129.0 (m), 128.6 (m), 128.2, 126.6, 126.5, 125.9, 123.2, 120.5, 117.4 (m), 61.6 (d, J = 24 Hz), 53.4, 52.7, 47.6, 45.2, 24.2, 10.4. **^{31}P NMR** ($CDCl_3$, 162 MHz): δ -19.6. **IR** (neat, cm^{-1}): ν = 2931, 1646, 1355, 1278, 1127, 887, 683. $[\alpha]_D^{21}$ = −17.0 (c = 0.5, $CHCl_3$). **HRMS-ESI** calcd for $C_{31}H_{32}N_2P$ [M-BAr$_F$]$^+$: 463.2298, found 463.2293.

Imidazolium salt 20e

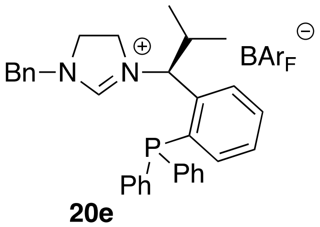

20e

Yield: 40%, (in three steps), colorless oil. **R$_f$** = 0.25 (DCM/Pentane, 3/1). **^{1}H NMR** ($CDCl_3$, 400 MHz): δ 7.72 (brs, 8H), 7.52 (brs, 4H), 7.42-7.36 (m, 13H), 7.20-7.16 (m, 4H), 7.06-7.04 (m, 3H), 5.2 (m, 1H), 4.12 (q, J = 31.6, 14.4 Hz, 2H), 3.62 (m, 1H), 3.52 (m, 2H), 3.30 (m, 1H), 2.46 (m, 1H), 0.91 (d, J = 6.4 Hz, 3H), 0.73 (d, J = 6.8 Hz). **^{13}C NMR** ($CDCl_3$, 100 MHz): δ 161.6 (dd, J = 99, 50 Hz), 155.10 (d, J = 6 Hz), 137.0, 136.8, 135.8, 134.8, 133.9, 133.7, 133.6, 130.2, 130.0 (m), 129.9 (m), 129.2-129.0 (m), 128.8, 128.6, 128.4, 128.1,126.7, 125.9, 123.2, 120.5, 117.5, 52.6, 47.5, 44.4, 29.7, 27.9, 19.7, 19.0. **^{31}P NMR** ($CDCl_3$, 162 MHz): δ -19.5. **IR** (neat, cm^{-1}): ν = 2968, 1645, 1355, 1278, 1125, 887. $[\alpha]_D^{21}$ = +11.6 (c = 1.0, $CHCl_3$). **HRMS-ESI** calcd for $C_{32}H_{34}N_2P$ [M-BAr$_F$]$^+$: 477.2454, found 477.2473.

Imidazolium salt 20f

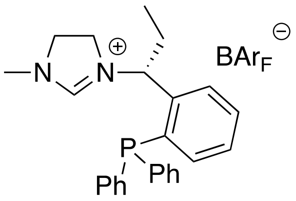

20f

Yield: 41%, white solid. **R$_f$** = 0.52 (DCM). **^{1}H NMR** ($CDCl_3$, 400 MHz): δ 7.74 – 7.68 (m, 9H), 7.55 (s, 4H), 7.47 – 7.31 (m, 9H), 7.22 – 7.12 (m, 4H), 7.09 (ddd, J = 7.6, 4.3, 1.5 Hz, 1H), 7.06 (s, 1H), 5.53 (q, J = 7.9 Hz, 1H), 3.55 (td, J = 12.2, 9.7 Hz, 1H), 3.41 – 3.17 (m, 2H), 3.07 (ddd, J = 12.9, 11.2, 9.5 Hz, 1H), 2.59 (s, 3H), 2.17 – 1.97 (m, 2H), 1.40 – 1.19 (m, 4H), 0.96 – 0.84 (m, 6H). **^{13}C NMR** ($CDCl_3$, 100 MHz): δ 161.6 (dd, J = 99.7, 49.9 Hz), 155.6 (d,

J = 3.9 Hz), 137.7 (d, J = 23.2 Hz), 137.1 (d, J = 14.0 Hz), 136.0, 134.9, 134.9 – 134.5 (m), 134.0 – 133.8 (m), 133.7 (d, J = 6.9 Hz), 130.3 (d, J = 14.1 Hz), 129.8 (d, J = 11.6 Hz), 129.5 – 128.3 (m), 126.6 (d, J = 4.6 Hz), 125.9, 123.2, 120.4, 117.8 – 117.3 (m), 61.5 (d, J = 24.9 Hz), 49.4, 45.3, 34.6, 24.2, 10.5. **^{31}P NMR** (162 MHz, CDCl3): δ –20.1. **IR** (neat, cm^{-1}): ν = 3072, 2978, 1658, 1610, 1436, 1355, 1277, 1124, 887. $[\alpha]_D^{21}$ = -18.0 (c = 1.0, $CHCl_3$). **ESI-HRMS** calcd for $C_{25}H_{28}N_2P$ $[M]^+$ 387.1990, found 387.2000.

Imidazolium salt 20g

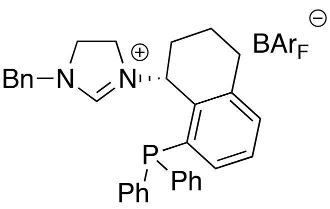

20g

Yield: 32%, (in three steps), white solid. **R$_f$** = 0.20 (DCM/Pentane, 3/1). **^{1}H NMR** ($CDCl_3$, 400 MHz): δ 7.73 (brs, 8H), 7.53 (brs, 4H), 7.46 (m, 2H), 7.36-7.26 (m, 10H), 7.19 (m, 1H), 7.10 (m, 2H), 7.02 (m, 1H), 6.95 (m, 2H), 6.72 (s, 1H), 5.47 (m, 1H), 4.04 (m, 2H), 3.80 (m, 2H), 3.34 (m, 2H), 2.80 (m, 2H), 2.02 (m, 1H), 1.91 (m, 2H), 1.32 (m, 1H). **^{13}C NMR** ($CDCl_3$, 100 MHz): δ 161.7 (dd, J = 100, 49 Hz), 155.4, 138.9 (d, J = 5 Hz), 137.26 (d, J = 15 Hz), 134.8, 134.4, 134.3, 134.1, 133.9, 133.5, 133.3, 133.1, 132.9, 131.7, 130.1 (m), 129.9 (m), 129. 7, 129.6, 129.5 (m), 129.1, 129.0, 128.9, 128.7 (m), 128.6, 128.1 (m), 127.8, 125.9, 123.2, 120.5, 117.5 (m), 55.0 (d, J = 24 Hz), 52.6, 47.6, 47.5, 47.4, 36.4, 31.3, 28.6, 26.2, 16.7. **^{31}P NMR** ($CDCl_3$, 162 MHz): δ -19.1. **IR** (neat, cm^{-1}): ν = 2948, 1645, 1355, 1278, 1125, 887, 682. $[\alpha]_D^{21}$ = −29.7 (c = 1.0, $CHCl_3$). **HRMS-ESI** calcd for $C_{32}H_{32}N_2P$ $[M\text{-}BAr_F]^+$: 475.2298, found 475.2298.

Imidazolium salt 20h

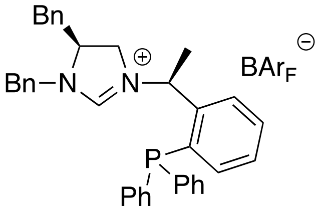

20h

Yield: 34%, (in three steps), colorless oil. **R$_f$** = 0.50 (DCM). **^{1}H NMR** ($CDCl_3$, 400 MHz): δ 7.73 (brs, 8H), 7.52 (brs, 4H), 7.44-7.01 (m, 23H), 6.86 (m, 2H), 5.47 (m, 1H), 4.42 (d, J = 14.8 Hz, 1H), 4.22 (d, J = 15.0 Hz, 1H), 4.13 (m, 1H), 3.65 (t, J = 12.0 Hz, 1H), 3.10 (dd, J = 12.0, 7.6 Hz, 1H), 2.96 (dd, J = 14.0, 4.8 Hz, 1H), 2.52 (dd, J = 13.6, 8.0 Hz, 1H), 1.45 (d, J = 6.8 Hz, 3H). **^{13}C NMR** ($CDCl_3$, 100 MHz): δ 161.6 (dd, J = 100, 49 Hz), 153.9, 139.1, 138.8, 136.4 (d, J = 15 Hz), 135.6, 134.8, 134.3, 134.0, 133.4, 133.2, 132.4, 130.4-129.7 (m), 129.5-128.2 (m), 126.0, 123.2, 117.5, 61.1, 55.9, 51.2, 37.5, 29.7,

18.2. **^{31}P NMR** (CDCl3, 162 MHz): δ-18.8. **IR** (neat, cm^{-1}): ν = 2926, 1641, 1355, 1278, 1127, 744, 682. $[\alpha]_D^{21}$ = +25.9 (*c* = 1.0, CHCl3). **HRMS-ESI** calcd for $C_{37}H_{36}N_2P$ [M-BAr$_F$]$^+$: 539.2611, found 539.2627.

Imidazolium salt 20i

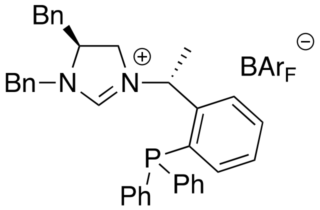

20i

Yield: 27%, (in three steps), colorless oil. **R$_f$** = 0.50 (DCM). **^{1}H NMR** (CDCl3, 400 MHz): δ7.73 (brs, 8H), 7.52 (brs, 4H), 7.48-7.21 (m, 15H), 7.21-7.01 (m, 7H), 7.01 (m, 1H), 6.86 (m, 2H), 5.47 (m, 1H), 4.42 (d, *J* = 14.8 Hz, 1H), 4.22 (d, *J* = 15.0 Hz, 1H), 4.13 (m, 1H), 3.65 (t, *J* = 12.0 Hz, 1H), 3.10 (dd, *J* = 12.0, 7.6 Hz, 1H), 2.96 (dd, *J* = 14.0, 4.8 Hz, 1H), 2.52 (dd, *J* = 13.6, 8.0 Hz, 1H), 1.45 (d, *J* = 6.8 Hz, 3H). **^{13}C NMR** (CDCl3, 100 MHz): δ161.7 (dd, *J* = 100, 49 Hz), 154.0, 138.7, 138.5, 136.4 (d, *J* = 15 Hz), 135.6, 134.8, 134.5, 134.0, 133.8 (m), 133.5 (m), 132.5, 130.5-129.8 (m), 129.6-128.4 (m), 126.0, 123.2, 120.5, 117.5, 61.0, 55.7, 50.9, 50.7, 37.5, 29.7, 18.0. **^{31}P NMR** (CDCl3, 162 MHz): δ-18.8. **IR** (neat, cm^{-1}): ν = 2926, 1641, 1355, 1278, 1127, 744, 682. $[\alpha]_D^{21}$ = -27.0 (*c* = 0.5, CHCl3). **HRMS-ESI** calcd for $C_{37}H_{36}N_2P$ [M-BAr$_F$]$^+$: 539.2611, found 539.2617.

Imidazolium salt 20j

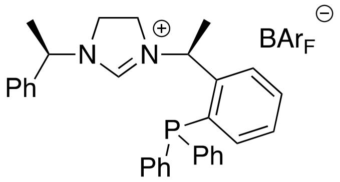

20j

Yield: 41% (in three steps), colorless oil, **R$_f$** = 0.40 (DCM). **^{1}H NMR** (CDCl3, 400 MHz): δ 7.76 – 7.66 (m, 8H), 7.52 (s, 4H), 7.49 – 7.28 (m, 13H), 7.22 – 7.12 (m, 4H), 7.09 – 7.01 (m, 3H), 5.70 (p, *J* = 6.9 Hz, 1H), 4.21 (q, *J* = 6.9 Hz, 1H), 3.66 – 3.52 (m, 1H), 3.44 – 3.29 (m, 2H), 3.28 – 3.16 (m, 1H), 1.58 (d, *J* = 6.9 Hz, 3H), 1.48 (d, *J* = 6.9 Hz, 3H). **^{13}C NMR** (CDCl3, 100 MHz): δ 161.7 (dd, *J* = 100, 50 Hz), 153.4 (d, *J* = 5 Hz), 139.0 (d, *J* = 22 Hz), 136.3 (d, *J* = 14 Hz), 135.9, 135.4, 134.8, 134.6 (d, *J* = 7 Hz), 134.2 (d, *J* = 6 Hz), 133.7, 133.5 (d, *J* = 3 Hz), 133.3, 130.5, 130.3, 130.1, 129.9, 129.8, 129.4 – 129.0 (m), 128.7 (m), 128.42 (m), 126.24, 126.16 (d, *J* = 5 Hz), 125.9, 123.2, 120.5, 117.7-117.4, 55.9, 55.73 (d, *J* = 24 Hz). 47.0, 45.3, 19.2, 18.0. **^{31}P NMR** (162 MHz, CDCl3): δ -19.3. **IR** (neat, cm^{-1}): ν = 3069, 2918, 1640, 1355, 1277, 1124. $[\alpha]_D^{21}$ = +

23.8 (c = 0.5, $CHCl_3$). **HRMS-ESI** calcd for $C_{31}H_{32}N_2P$ [M-BAr$_F$]$^+$: 463.2298, found 463.2282.

Imidazolium salt 20k

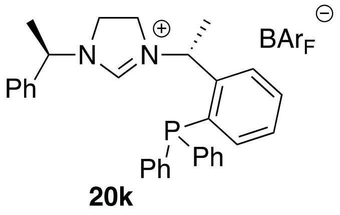

Yield: 26% (in three steps), colorless oil. **R$_f$** = 0.40 (DCM). **^{1}H NMR** ($CDCl_3$, 400 MHz): δ 7.80 (m, 8H), 7.58 (s, 4H), 7.48 – 7.28 (m, 13H), 7.28-7.24 (m, 2H), 7.21-7.17 (m, 2H), 7.13 – 7.10 (m, 3H), 5.65 (p, J = 6.8 Hz, 1H), 4.28 (q, J = 6.8 Hz, 1H), 3.72 – 3.69 (m, 1H), 3.54 – 3.35 (m, 3H), 1.64 (d, J = 6.8 Hz, 3H), 1.49 (d, J = 6.8 Hz, 3H). **^{13}C NMR** ($CDCl_3$, 100 MHz): δ 161.7 (dd, J = 100, 50 Hz), 153.3 (d, J = 5 Hz), 139.0 (d, J = 22 Hz), 136.3 (d, J = 14 Hz), 135.8, 135.5, 134.8, 134.5 (d, J = 7 Hz), 134.3 (d, J = 7 Hz), 134.0, 133.8, 133.5, 133.3, 130.5, 130.3, 130.1, 129.9, 129.8, 129.3 – 129.1 (m), 128.7(m), 128.64-128.5 (m), 126.3, 126.2 (m), 125.9, 123.2, 120.5, 117.5 (m), 59.0, 56.0 (d, J = 24 Hz). 53.4, 47.0, 45.9, 19.2, 18.0. **^{31}P NMR** (162 MHz, CDCl3): δ -19.0. **IR** (neat, cm^{-1}): ν = 3059, 2927, 1640, 1355, 1278, 1126, 887, 742. $[\alpha]_D^{21}$ = -18.5 (c = 0.5, $CHCl_3$). **HRMS-ESI** calcd for $C_{31}H_{32}N_2P$ [M-BAr$_F$]$^+$: 463.2298, found 463.2304.

Synthesis of chiral NHC-phosphine iridium complexes Ir-1 to 11

Compound **20** (1.0 equiv.) was dissolved in dry THF (0.05M) under argon, followed by addition of $[Ir(COD)Cl]_2$ (0.5 equiv.) and KO^{t}Bu (1.05 equiv.) and the mixture was allowed to stir at room temperature for 3 h. The solvent was removed under vacuum and the residue was purified by flash column chromatography on silica gel (DCM/pentane, 1/1 to 3/1) to obtain catalysts **Ir-1** to **Ir-11** as red solids.

NHC-phosphine iridium complex Ir-1

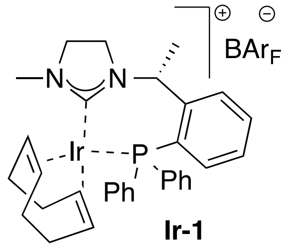

Yield: 58%, red solid. **R$_f$** = 0.84 (DCM). **^{1}H NMR** ($CDCl_3$, 400 MHz): δ 7.75 – 7.68 (m, 9H), 7.57 – 7.23 (m, 19H), 7.12 – 7.07 (m, 2H), 4.94 – 4.89 (m, 1H), 4.39 – 4.33 (m, 1H), 4.08 – 4.03 (m, 1H), 3.83 – 3.77 (m, 1H), 3.45 – 3.38 (m, 1H), 3.22 (q, 1H, J = 9.8 Hz), 2.92 – 2.84 (m, 1H), 2.77 – 2.70 (m, 1H), 2.49 – 2.29 (m, 4H), 2.24 (s, 3H), 2.11 – 2.00 (m, 2H), 1.92 – 1.83 (m, 1H), 1.71 – 1.79 (m, 1H), 1.69 (d, 3H, J = 7.1 Hz), 1.31 – 1.23 (m, 1H). **^{13}C NMR** ($CDCl_3$, 100 MHz): δ 198.1 (d, J = 10.7 Hz), 161.7 (q, J = 49.8 Hz), 144.3 (d, J =

12.2 Hz), 139.3 (d, J = 2.4 Hz), 134.8, 133.1 (d, J = 11.2 Hz), 131.85, 131.82, 131.7, 131.5, 130.9 – 131.1 (m), 128.4 – 129.4 (m), 128.3, 127.8, 126.8 (d, J = 8.5 Hz), 125.9, 123.2, 120.5, 117.5 – 117.4 (m), 89.0 (d, J = 9.6 Hz), 84.6 (d, J = 13.2 Hz), 80.5, 78.7, 55.8 (d, J = 7.9 Hz), 51.4, 42.6, 35.6, 34.6, 34.0 (d, J = 3.7 Hz), 29.7, 28.45 (d, J = 1.7 Hz), 28.1 (d, J = 1.8 Hz), 16.0. **^{31}P NMR** (CDCl$_3$, 162 MHz): δ 8.29. **IR** (neat, cm^{-1}) ν = 2956, 2888, 1610, 1531, 1354, 1277, 1128. $[\alpha]_D^{21}$ = - 64.0 (c = 0.1, CHCl$_3$) **HRMS-ESI** calcd for $C_{32}H_{37}IrN_2P$ [M-BAr$_F$]$^+$: 673.2320 found 673.2291.

NHC-phosphine iridium complex Ir-2

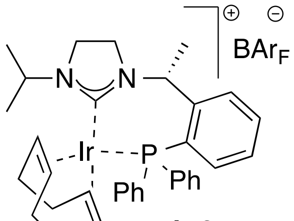

Ir-2

Yield: 41%, red solid. **R$_f$** = 0.84 (DCM). **^{1}H NMR** (CDCl$_3$, 400 MHz): δ 7.72 – 7.69 (m, 8H), 7.57 – 7.46 (m, 11H), 7.37 – 7.29 (m, 4H), 7.25 – 7.23 (m, 1H), 7.15 – 7.10 (m, 1H), 7.07 – 7.02 (m, 2H), 5.02 – 4.95 (m, 1H), 4.54 – 4.45 (m, 1H), 3.98 – 3.91 (m, 2H), 3.58 – 3.52 (m, 1H), 3.49 – 3.43 (m,1H), 3.24 – 3.16 (q, 1H, J = 10.8 Hz), 3.01 – 2.94 (m, 1H), 2.79 (m, 1H), 2.60 – 2.30 (m, 4H), 2.12 – 2.04 (m, 1H), 1.98 – 1.90 (m, 1H), 1.82 – 1.75 (m, 1H), 1.71 (d, 3H, J = 7.12 Hz), 1.65-1.57 (m 1H), 1.28-1.20 (m, 1H), 0.96 (d, 3H, J = 7.12 Hz), -0.09 (d, J = 6.6 Hz, 3H). **^{13}C NMR** (CDCl$_3$, 100 MHz): δ 196.1 (d, J =10 Hz), 161.6 (dd, J = 100, 50 Hz), 144.0 (d, J = 12 Hz), 139.7 (d, J = 2.0 Hz), 134.7, 133.3 (d, J = 11 Hz), 131.84, 131.82, 131.7, 131.6 (d, J = 2 Hz), 130.8, 130.7 (d, J = 2 Hz), 130.3, 129.5, 129.4, 129.3 – 129.2 (m), 129.0 – 128.9 (m), 128.8 (d, J = 3 Hz), 128.8 – 128.6 (m), 128.5 (d, J = 3 Hz), 128.4 – 128.2 (m), 126.9 (d, J = 8 Hz), 125.8, 123.1, 120.4, 117.4 – 117.5 (m), 89.4 (d, J = 9 Hz), 84.0 (d, J = 14 Hz), 79.1, 78.6, 77.2, 56.0 (d, J = 8 Hz), 50.4, 42.3, 41.9, 35.38, 35.36, 34.7 (d, J = 4 Hz), 29.7, 27.9 – 27.1 (m), 21.7, 18.8, 16.2. **^{31}P NMR** (CDCl$_3$, 162 MHz): δ 6.3. **IR** (neat, cm^{-1}): ν = 1354, 1277, 1125. $[\alpha]_D^{21}$ = – 87.0 (c = 0.1, CHCl$_3$). **HRMS-ESI** calcd for $C_{34}H_{41}IrN_2P$ [M-BAr$_F$]$^+$: 701.2631, found 701.2618.

NHC-phosphine iridium complex Ir-3

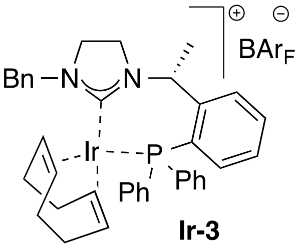

Ir-3

Yield: 52%, red solid. **R$_f$** = 0.80 (DCM). **^{1}H NMR** (CDCl$_3$, 400 MHz): δ 7.76 (brs, 8H), 7.59-7.47 (m, 9H), 7.47-7.22 (m, 11), 6.83 (m, 1H), 6.83 (m, 2H), 5.09 (m, 1H), 4.87 (d, J

= 16 Hz), 4.45 (m, 1H), 4.08 (m, 1H), 3.92 (m, 1H), 3.90 (m, 1H), 3.52 (q, *J* = 8.8 Hz, 1H), 3.29 (q, *J* = 8.4 Hz, 1H), 3.04 (m, 1H), 2.66 (m, 1H), 2.63 (m, 5H), 2.05 (m, 2H), 1.75 (m, 5H), 1.29 (m, 1H). **^{13}C NMR** ($CDCl_3$, 100 MHz): *δ* 198.7 (d, *J* = 10 Hz), 162.0 (dd, *J* = 100, 50 Hz), 144.3 (d, *J* = 12 Hz), 139.4 (d, *J* = 2 Hz), 134.8, 134.5, 133.2 (d, *J* = 11 Hz), 131.9 (m), 131.8, 131.7, 131.2 (m), 131.1, 131.0, 129.3, 129.2, 129.1 (m), 128.6 (m), 128.4, 128.2, 127.7, 126.7, 126.6, 126.4, 125.9, 123.2, 120.5, 117.5 (m), 88.7 (d, *J* = 9 Hz), 85.4 (d, *J* = 13 Hz), 80.7, 79.2, 56.2, 56.1, 52.9, 48.7, 42.7, 35.0, 34.2, 28.3, 29.8, 27.7, 15.8. **^{31}P NMR** ($CDCl_3$, 162 MHz): *δ* 7.6. **IR** (neat, cm^{-1}): ν = 2925, 1610, 1505, 1354, 1277, 1125, 887. $[\alpha]_D^{21}$ = -56.0 (*c* =0.1, $CHCl_3$). **HRMS-ESI** calcd for $C_{38}H_{41}IrN_2P$ $[M-BAr_F]^+$: 749.2633, found 749.2617.

NHC-phosphine iridium complex Ir-4

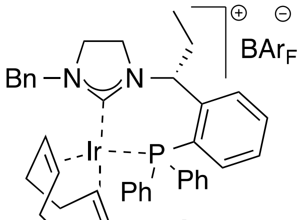

Ir-4

Yield: 59%, red solid. **R_f** = 0.80 (DCM). **^{1}H NMR** ($CDCl_3$, 400 MHz): *δ* 7.72 (brs, 8H), 7.57-7.40 (m, 9H), 7.40-7.26 (m, 10H), 7.10 (m, 1H), 6.84-6.77 (m, 3H), 5.06 (m, 1H), 4.83 (d, *J* = 15.6 Hz, 1H), 4.44 (m, 1H), 4.01 (m, 1H), 3.92 (m, 1H), 3.42 (m, 1H), 3.30 (m, 1H), 3.01 (m, 1H), 2.65 (m, 1H), 2.64-2.30 (m, 6H), 2.20-1.97 (m, 4H), 1.92-1.74 (m, 2H), 1.25-1.12 (m, 3H). **^{13}C NMR** ($CDCl_3$, 100 MHz): *δ* 199.3 (d, *J* = 11 Hz), 161.9 (dd, *J* = 100, 50 Hz), 144.1 (d, *J* = 12 Hz), 139.5, 134.8, 134.3, 133.2 (d, *J* = 11 Hz), 131.9, 131.8, 131.7, 131.2 (m), 131.1 (m), 129.6, 129.3, 129.2 (m), 129.1-129.0 (m), 128.7-128.5 (m), 128.1, 127.6, 127.0 (d, *J* = 8 Hz), 126.3, 125.9, 123.2, 120.5, 117.4 (m), 87.9 (d, *J* = 10 Hz), 86.1, 85.9, 80.4, 79.1, 62.7, 62.6, 52.9, 48.8, 42.4, 34.5, 33.9, 33.8, 34.5, 33.9, 33.8, 29.7, 28.6, 28.0, 22.3, 11.4. **^{31}P NMR** ($CDCl_3$, 162 MHz): *δ* 6.7. **IR** (neat, cm^{-1}): ν = 2925, 1610, 1505, 1354, 1277, 1124, 887. $[\alpha]_D^{21}$ = -47.0 (*c* =0.1, $CHCl_3$). **HRMS-ESI** calcd for $C_{39}H_{43}IrN_2P$ $[M-BAr_F]^+$: 763.2788, found 763.2799.

NHC-phosphine iridium complex Ir-5

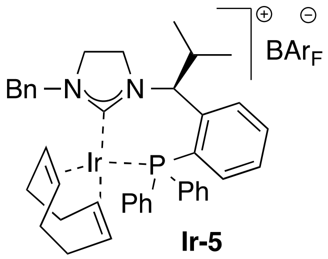

Ir-5

Yield: 42%, red solid. **R_f** = 0.8 (DCM). **^{1}H NMR** ($CDCl_3$, 400 MHz): *δ* 7.40 (m, 8H), 7.53-7.24 (m, 19H), 7.15 (m, 1H), 6.76 (m, 12), 6.66 (dd, *J* = 11.2, 1.6 Hz, 1H), 5.04 (m, 1H), 4.70 (d, *J* = 16 Hz, 1H), 4.46 (m, 1H), 4.01-3.92 (m, 2H), 3.45 (m, 1H), 3.34 (m, 1H), 3.10 (m, 1H), 2.75

(m, 1H), 2.64 (d, *J* = 16 Hz), 2.42-2.36 (m, 5H), 2.20-1.99 (m, 2H), 1.89-1.81 (m, 2H), 1.79 (m, 4H), 1.14 (d, *J* = 6.4 Hz, 3H). **^{13}C NMR** ($CDCl_3$, 100 MHz): δ 199.3 (d, *J* = 10 Hz), 161.6 (dd, *J* = 100, 50 Hz), 142.0 (d, *J* = 12 Hz), 139.3 (d, *J* = 3 Hz), 134.8, 134.1, 133.2, 133.1, 131.9, 131.8, 131.6, 131.5, 131.2, 130.9, 130.5, 130.0, 129.3-128.7 (m), 127.9, 127.5, 127.4 (m), 126.3, 125.9, 123.2, 120.5, 117.4 (m), 87.1 (d, *J* = 10 Hz), 85.9 (d, *J* = 13 Hz), 80.0, 79.1, 68.2, 68.1, 52.7, 49.0, 42.7, 34.0, 33.3 (m), 29.0, 28.5, 26.6, 20.6, 20.4. **^{31}P NMR** ($CDCl_3$, 162 MHz): δ 5.5. **IR** (neat, cm^{-1}): ν = 2927, 1611, 1355, 1277, 1125, 887. $[\alpha]_D^{21}$ = -54.0 (*c* =0.1, $CHCl_3$). **HRMS-ESI** calcd for $C_{40}H_{45}IrN_2P$ [M-BAr_F]$^+$: 777.2946, found 777.2947.

NHC-phosphine iridium complex Ir-6

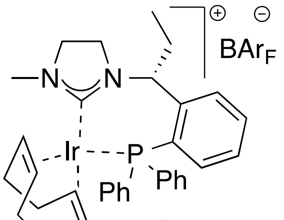

Ir-6

Yield: 74%, red solid. **R$_f$** = 0.84 (DCM). **^{1}H NMR** ($CDCl_3$, 400 MHz): δ 7.75 – 7.66 (m, 9H), 7.57 – 7.22 (m, 15H), 7.07 (ddd, *J* = 11.1, 7.9, 1.4 Hz, 1H), 6.73 (ddd, *J* = 11.3, 4.0, 1.7 Hz, 1H), 4.92 (m, 1H), 4.45 – 4.36 (m, 1H), 4.00 (m, 1H), 3.83 (m, 1H), 3.39 – 3.22 (m, 2H), 2.95 – 2.86 (m, 1H), 2.78 (m, 1H), 2.51 – 2.27 (m, 5H), 2.21 (s, 3H), 2.16 – 2.01 (m, 3H), 1.89 (m, 1H), 1.77 (m, 1H), 1.15(t, *J* = 7.2 Hz, 3H). **^{13}C NMR** ($CDCl_3$, 100 MHz): δ 198.7 (d, *J* = 10.7 Hz), 161.7 (dd, *J* = 99.6, 49.8 Hz), 144.1 (d, *J* = 12.2 Hz), 139.3 (d, *J* = 2.4 Hz), 134.8, 133.0 (d, *J* = 11.0 Hz), 131.8, 131.7, 131.4, 131.06, 131.04, 131.01, 130.9, 129.7, 129.4 – 128.3 (m), 128.2, 127.7, 127.0 (d, *J* = 8.4 Hz), 125.9, 123.2, 120.5, 117.6 – 117.2 (m), 88.3 (d, *J* = 9.9 Hz), 85.3 (d, *J* = 12.8 Hz), 80.2, 78.5, 62.3 (d, *J* = 8.0 Hz), 51.5, 42.3, 35.5, 34.2 (d, *J* = 1.5 Hz), 33.7 (d, *J* = 3.7 Hz), 28.7 (d, *J* = 1.7 Hz), 28.3 (d, *J* = 2.1 Hz), 22.4, 11.3. **^{31}P NMR** (162 MHz, CDCl3): δ 7.4. **IR** (neat, cm^{-1}): ν = 2927, 1609, 1529, 1353, 1276, 1130, 886. $[\alpha]_D^{21}$ = -54.0 (*c* = 0.1, $CHCl_3$). **HRMS-ESI** calcd for $C_{33}H_{39}IrN_2P$ [M]$^+$ 687.2476, found 687.2473.

NHC-phosphine iridium complex Ir-7

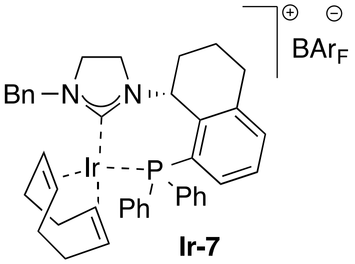

Ir-7

Yield: 38%, red solid. **R$_f$** = 0.7 (DCM). **^{1}H NMR** ($CDCl_3$, 400 MHz): δ 7.73 (m, 8H), 7.60-7.23 (m, 18H), 7.12 (m, 1H), 6.95 (m, 1H), 6.84 (m, 2H), 5.07 (m, 1H), 4.98 (d, *J* = 15.6 Hz, 1H), 4.42 (m, 1H), 4.02 (m, 1H), 3.87 (m, 1H), 3.63 (q, *J* = 9.2 Hz, 1H), 3.29-3.17 (m,

2H), 2.91-2.81 (m, 2H), 2.57-2.40 (m, 3H), 2.17 (m, 1H), 2.17-1.70 (m, 7H). **^{13}C NMR** ($CDCl_3$, 100 MHz): *δ* 198. 8 (d, *J* = 10 Hz), 161.7 (dd, *J* = 100, 50 Hz), 140.1 (d, *J* =8 Hz), 139.2 (d, *J* = 12 Hz), 137.7, 134.8, 134.4, 133.5, 133.4, 133.2, 133.0, 132.2 (m), 131.8, 131.7, 131.6, 131.5, 131.4, 131.2, 130.9, 129.3, 129.2-129.2 (m), 129.0-128.8 (m), 128.7, 128.6, 128.5(m), 128.4-127.6 (m), 127.8, 127.6, 126.4, 125.6, 123.5, 121.3, 117.4 (m), 87.9 (d, *J* = 10 Hz), 85.3 (d, *J* =13 Hz), 80.9, 79.0, 55.7, 55.6, 53, 49.1, 47.7, 44.9, 40.9, 35.1, 34.4, 34.3, 30.2, 28.2, 28.0, 27.7, 19.5. **^{31}P NMR** ($CDCl_3$, 162 MHz): *δ* 6.7. **IR** (neat, cm^{-1}): ν = 2928, 1611, 1454, 1354, 1277, 1125, 887. $[\alpha]_D^{21}$ = -46.0 (*c* =0.1, $CHCl_3$). **HRMS-ESI** calcd for $C_{40}H_{43}IrN_2P$ [M-BAr_F]$^+$: 775.2788, found 775.2794.

NHC-phosphine iridium complex Ir-8

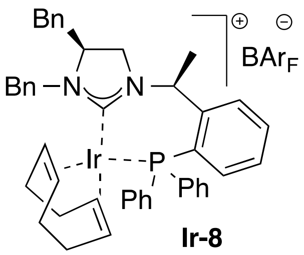

Ir-8

Yield: 58%, red solid. **R_f** = 0.45 (DCM/Pentane, 1/1). **^{1}H NMR** ($CDCl_3$, 400 MHz): *δ* 7.77 (brs, 8H), 7.68 (brs, 4H), 7.66-7.12 (m, 20H), 6.91 (m, 2H), 6.45 (m, 2H), 5.16-5.13 (m, 2H), 4.48 (m, 1H), 4.05 (m, 1H), 3.86-3.70 (m, 2H), 3.43 (t, *J* = 13.6 Hz, 1H), 3.13 (d, *J* = 21.2 Hz, 1H), 2.62-2.35 (m, 6H), 2.02 (m, 3H), 1.80-1.64 (m, 4H), 1.30 (m, 2H). **^{13}C NMR** ($CDCl_3$, 100 MHz): *δ* 198.7 (d, *J* = 10.5 Hz). 162.0 (dd, *J* = 99.0, 49.0 Hz), 144.4 (d, *J* = 12.0 Hz), 139.8, 135.0, 134.9, 134.8, 134, 6, 133.7, 132.4, 123.2, 132.0, 131.4, 131.3, 131.2, 130.2-129.0 (m), 128.9-128.1 (m), 127.6-127.5 (m), 126.8, 126.6, 123.0, 119.4, 117.7, 89.6 (d, *J* = 9.4 Hz), 85.5 (d, *J* = 13.4 Hz), 80.9, 79.5, 59.9, 56.8, 56.7, 50.9, 48.3, 37.8, 35.5, 34.8, 34.7, 28.4, 27.7, 16.3. **^{31}P NMR** ($CDCl_3$, 162 MHz): *δ* 8.9. **IR** (neat, cm^{-1}): ν = 2928, 1610, 1437, 1354, 1277, 1124, 887, 744. $[\alpha]_D^{21}$ = +86.0 (*c* = 0.1, $CHCl_3$). **HRMS-ESI** calcd for $C_{45}H_{47}IrN_2P$ [M-BAr_F]$^+$: 839.3103, found 839.3081.

NHC-phosphine iridium complex Ir-9

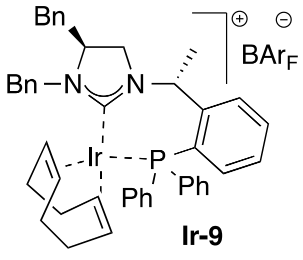

Ir-9

Yield: 42%, red solid. **R_f** = 0.45 (DCM/Pentane, 1/1). **^{1}H NMR** ($CDCl_3$, 400 MHz): *δ* 7. 77 (brs, 8H), 7.68-7.12 (m, 20H), 6.88 (m, 2H), 6.51 (m, 2H), 4.85 (m, 1H), 4.60 (m, 2H), 4.10 (m, 2H), 3.74 (m, 2H), 3.38 (m, 2H), 2.96 (m, 1H), 2.61-2.47 (m, 6H), 2.10-1.71 (m, 3H), 1.68 (m, 4H). **^{13}C NMR** ($CDCl_3$, 100 MHz): *δ* 199.4 (d, *J* = 10.2 Hz),

161.9 (dd, J = 99.0, 49.0 Hz), 143.9 (d, J = 12.3 Hz), 139.5, 135.2, 135.0, 134.0, 133.9, 133.8, 132.1, 132.0, 131.9, 131.7-130.1 (m), 129.7-128.3 (m), 127.5, 127.4, 126.9, 126.8, 126.5, 122.9, 119.3, 117.6, 89.3 (d, J = 9.1 Hz), 84.7 (d, J = 13.4 Hz), 81.3, 80.3, 68.1, 61. 8, 56.3, 52.9, 47.8, 38.3, 35.1, 34.6, 34.5, 28.3, 27.6, 25.7, 16.0. **^{31}P NMR** ($CDCl_3$, 162 MHz): δ 7.1. **IR** (neat, cm^{-1}): ν = 2928, 1610, 1437, 1354, 1277, 1124, 887, 744. $[\alpha]_D^{21}$ = −42.0 (c = 0.1, $CHCl_3$). **HRMS-ESI** calcd for $C_{45}H_{47}IrN_2P$ $[M-BAr_F]^+$: 839.3103, found 839.3090.

NHC-phosphine iridium complex Ir-10

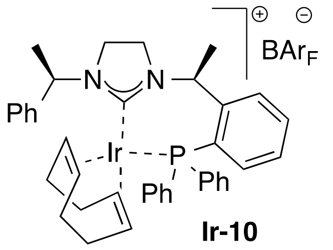

Ir-10

Yield: 41%, red solid. **R$_f$** = 0.7 (DCM). **^{1}H NMR** ($CDCl_3$, 400 MHz): δ 7. 78 (brs, 8H), 7.69-7.45 (m, 10H), 7.44-7.25 (m, 8H), 7.24-7.25 (m, 2H), 7.20-6.90 (m, 3H), 6.40-6.36 (m, 1H), 5.50-5.41 (m, 1H), 5.21-5.10 (m, 1H), 4.47-4.39 (m, 1H), 4.23-4.09 (m, 1H), 3.51-3.27 (m, 3H), 3.20-2.89 (m, 2H), 2.49-2.15 (m, 4H), 1.75 (d, J = 7.1 Hz, 3H), 1.60 (d, J = 6.9 Hz, 3H), 1.81-1.49 (m, 4H). **^{13}C NMR** ($CDCl_3$, 100 MHz): δ 201.05 (d, J = 10.1 Hz), 161.7 (dd, J = 99.7, 49.8 Hz), 143.0 (d, J = 11.8 Hz), 137.8, 137.4-137.3 (m), 134.8, 134.4 (d, J = 12.7 Hz), 132.2, 132.1, 131.8 – 131.7 (m), 130.9 (d, J = 2.1 Hz), 129.91 (d, J = 6.5 Hz), 129.5 – 128.3 (m), 127.3, 126.9, 126.6 (d, J = 8.1 Hz), 125.9, 125.1, 123.2, 120.5, 117.6 – 117.3 (m), 88.96 (d, J = 8.6 Hz), 82.56 (d, J = 14.7 Hz), 81.2, 80.1, 57.4, 56.15 (d, J = 10.3 Hz), 44.4, 42.4, 35.8 – 35.7 (m), 35.26 (d, J = 4.3 Hz), 29.7, 27.61 (d, J = 1.9 Hz), 26.7 – 26.6 (m), 20.1, 16.1. **^{31}P NMR** (162 MHz, CDCl3): δ 3.9. **IR** (neat, cm^{-1}): ν = 2926, 1610, 1437, 1354, 1277, 1125, 887. $[\alpha]_D^{21}$ = + 63.0 (c = 0.5, $CHCl_3$). **HRMS-ESI** calcd for $C_{39}H_{43}IrN_2P$ $[M-BAr_F]^+$: 763.2788, found 763.2786.

NHC-phosphine iridium complex Ir-11

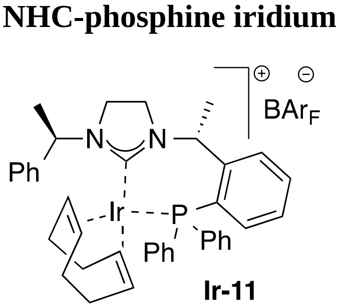

Ir-11

Yield: 36%, red solid. **R$_f$** = 0.7 (DCM). **^{1}H NMR** ($CDCl_3$, 400 MHz): δ 7.75 (m, 8H), 7.58-7.50 (m, 10H), 7.48-7.19 (m, 8H), 7.16-7.11 (m, 3H), 6.93 (m, 2H), 5.07 (m, 1H), 4.99 (m, 1H), 4.03 (m, 2H), 3.62 (m, 2H), 3.45 (m, 1H), 3.06 (m, 2H), 2.49-2.34 (m, 4H), 1.91-1.58 (m, 8H), 0.41 (d, J = 6.4 Hz, 3H). **^{13}C NMR** ($CDCl_3$, 100 MHz): δ 198.2 (d, J = 11 Hz), 161.9 (dd,

J = 100, 50 Hz), 144.2 (d, J = 12 Hz), 141.2, 139.9, 134.8, 133.3 (d, J = 11 Hz), 131.9 (m), 131.8, 131.7 (m), 131.5, 130.9, 130.8, 129.6, 129.5, 129.4 (m), 129.1, 129.0 (m), 128.9, 128.7-128.6 (m), 128.4 (m), 127.9, 126.8 (m), 125.9, 124.8, 123.2, 120.5, 117.5 (m), 89.5 (d, J = 9 Hz), 85.6, 85.5, 79.7, 78.9, 67.9, 57.0, 56.3 (d, J = 9 Hz), 44.7, 42.2, 35.4, 34.8, 29.7, 27.7, 27.0, 25.6, 18.5, 15.9, 14.1. **^{31}P NMR** ($CDCl_3$, 162 MHz): δ 6.6. **IR** (neat, cm^{-1}): ν = 3054, 2987, 1609, 1422, 1355, 1265, 1127, 896, 738. $[\boldsymbol{\alpha}]_{\mathbf{D}}^{\mathbf{21}}$ = -91.7 (c = 0.5, $CHCl_3$). **HRMS-ESI** calcd for $C_{39}H_{43}IrN_2P$ $[M\text{-}BAr_F]^+$ 763.2788, found 763.2835.

Separation of chiral products and chromatograms

Product	*ee* (%)	Retention time		Separation method
		T_{R1}/min	T_{R2}/min	
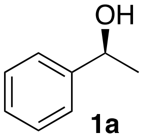 **1a**	94 (*S*)	48.98	51.02	GC-MS: Chiraldex BDM column, 50-110 °C, 1 °C/min
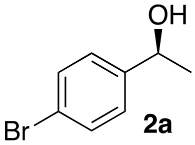 **2a**	94 (*S*)	73.21	74.85	GC-MS: Chiraldex BDM column, 50-170 °C, 1 °C/min
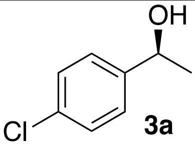 **3a**	94 (*S*)	62.65	64.90	GC-MS: Chiraldex BDM column, 50-170 °C, 1 °C/min
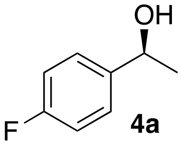 **4a**	94 (*S*)	42.70	44.99	GC-MS: Chiraldex BDM column, 50-110 °C, 1 °C/min
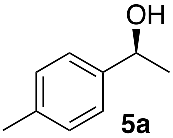 **5a**	94 (*S*)	45.14	48.46	GC-MS: Chiraldex BDM column, 50-110 °C, 1 °C/min
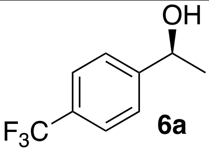 **6a**	95 (*S*)	45.18	48.44	GC-MS: Chiraldex BDM column, 50-110 °C, 1 °C/min
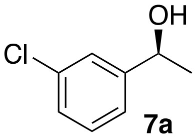 **7a**	95 (*S*)	64.97	66.44	GC-MS: Chiraldex BDM column, 50-170 °C, 1 °C/min
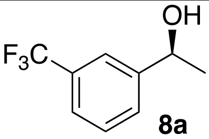 **8a**	96 (*S*)	45.79	47.00	GC-MS: Chiraldex BDM column, 50-110 °C, 1 °C/min
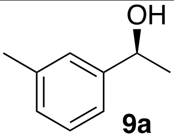 **9a**	89 (*S*)	10.33	11.50	SFC: IF column, 5% MeOH, 2 ml/min

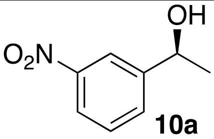 **10a**	90 (*S*)	14.48	16.61	SFC: OJH column, 5% MeOH, 2 ml/min
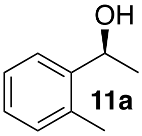 **11a**	92 (*S*)	54.95	60.34	GC-MS: Chiraldex BDM column, 50-170 °C, 1 °C/min
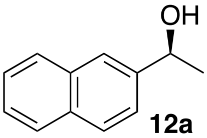 **12a**	90 (*S*)	18.36	20.78	SFC: ID column, 5% MeOH, 2 ml/min
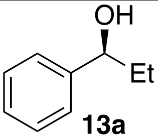 **13a**	93 (*S*)	50.18	51.11	GC-MS: Chiraldex BDM column, 50-110 °C, 1 °C/min

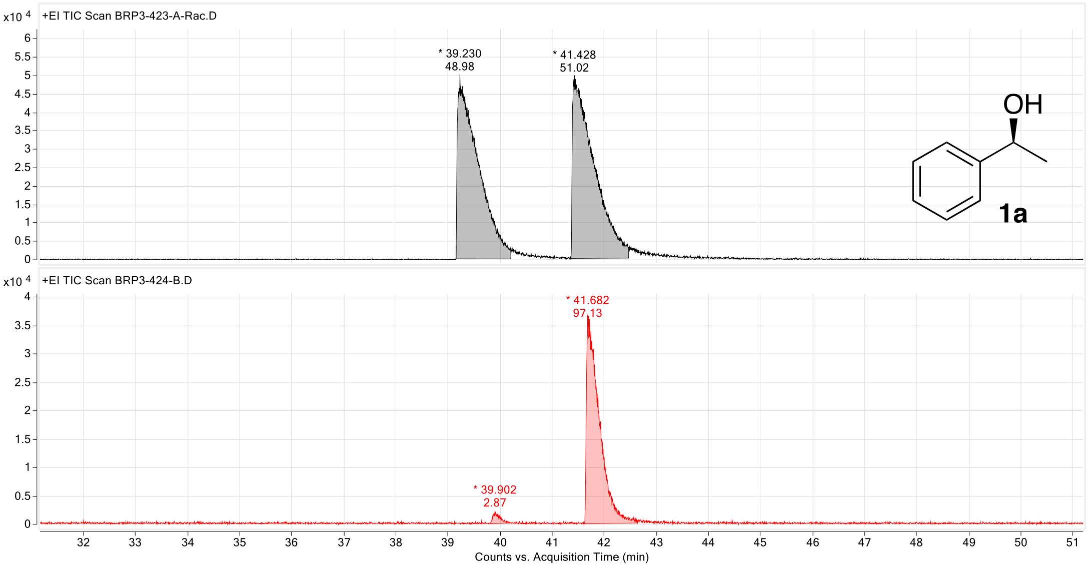

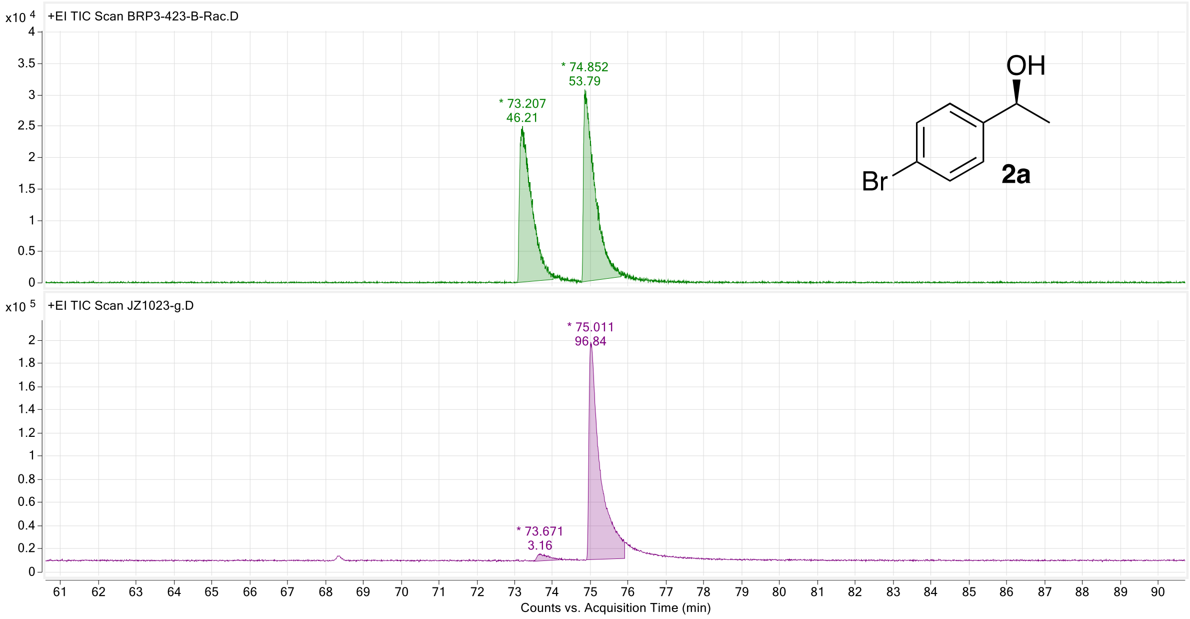
+EI TIC Scan BRP3-423-B-Rac.D
x10 4
* 73.207
46.21
* 74.852
53.79
OH
Br
2a
+EI TIC Scan JZ1023-g.D
x10 5
* 75.011
96.84
* 73.671
3.16
Counts vs. Acquisition Time (min)

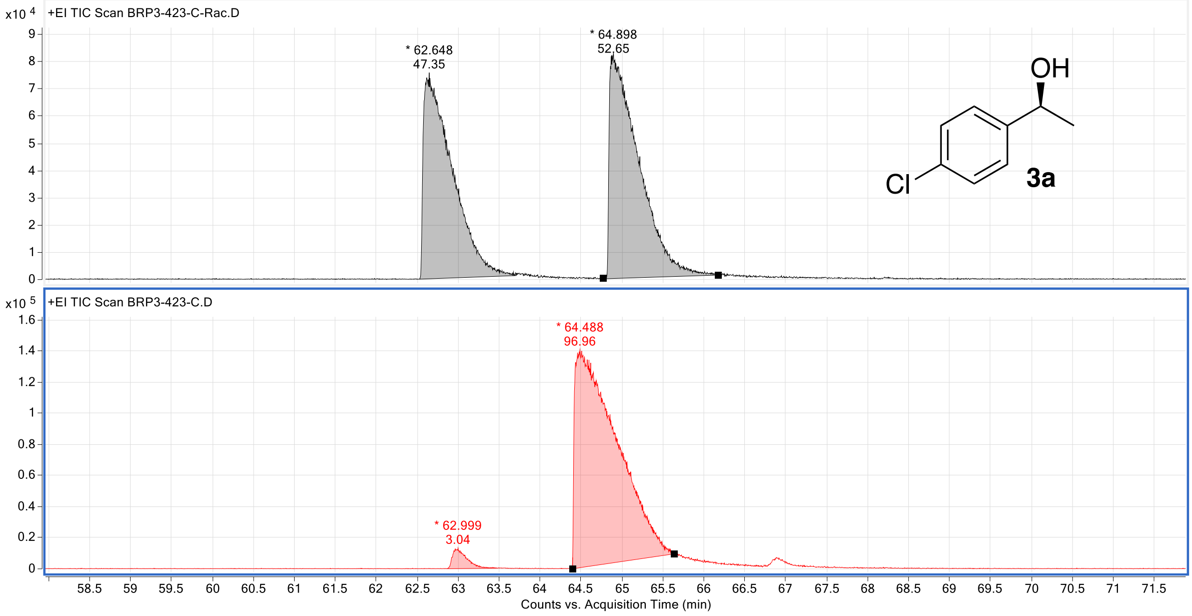
+EI TIC Scan BRP3-423-C-Rac.D
x10 4
* 62.648
47.35
* 64.898
52.65
OH
Cl
3a
+EI TIC Scan BRP3-423-C.D
x10 5
* 64.488
96.96
* 62.999
3.04
Counts vs. Acquisition Time (min)

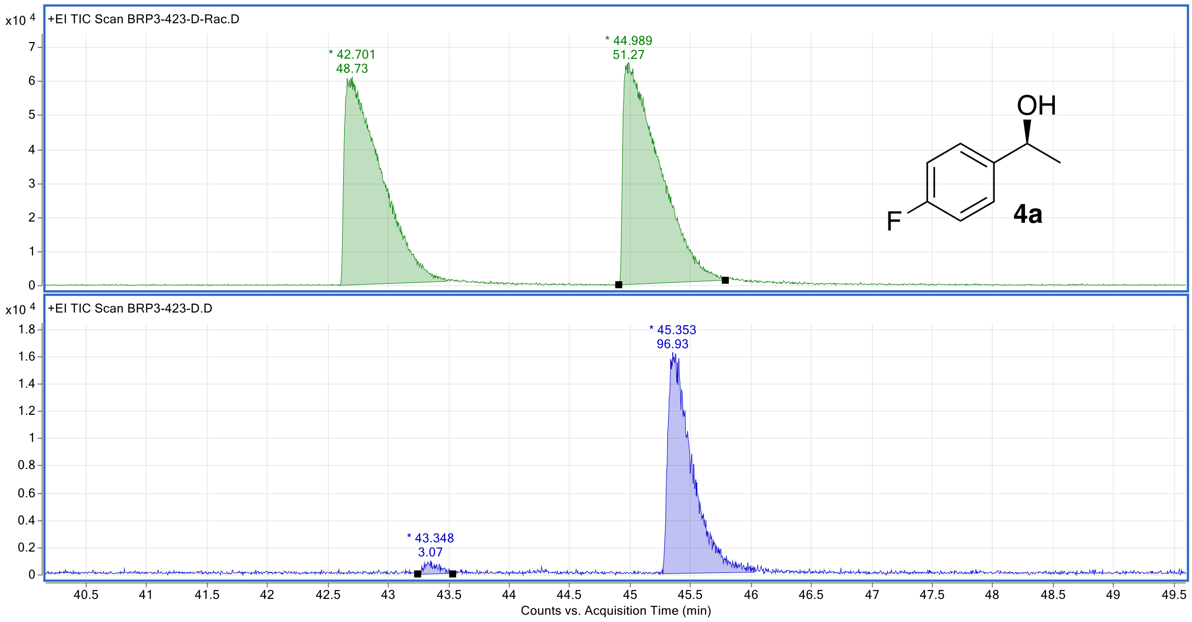
+EI TIC Scan BRP3-423-D-Rac.D
x10 4
* 42.701
48.73
* 44.989
51.27
OH
F
4a
+EI TIC Scan BRP3-423-D.D
x10 4
* 45.353
96.93
* 43.348
3.07
Counts vs. Acquisition Time (min)

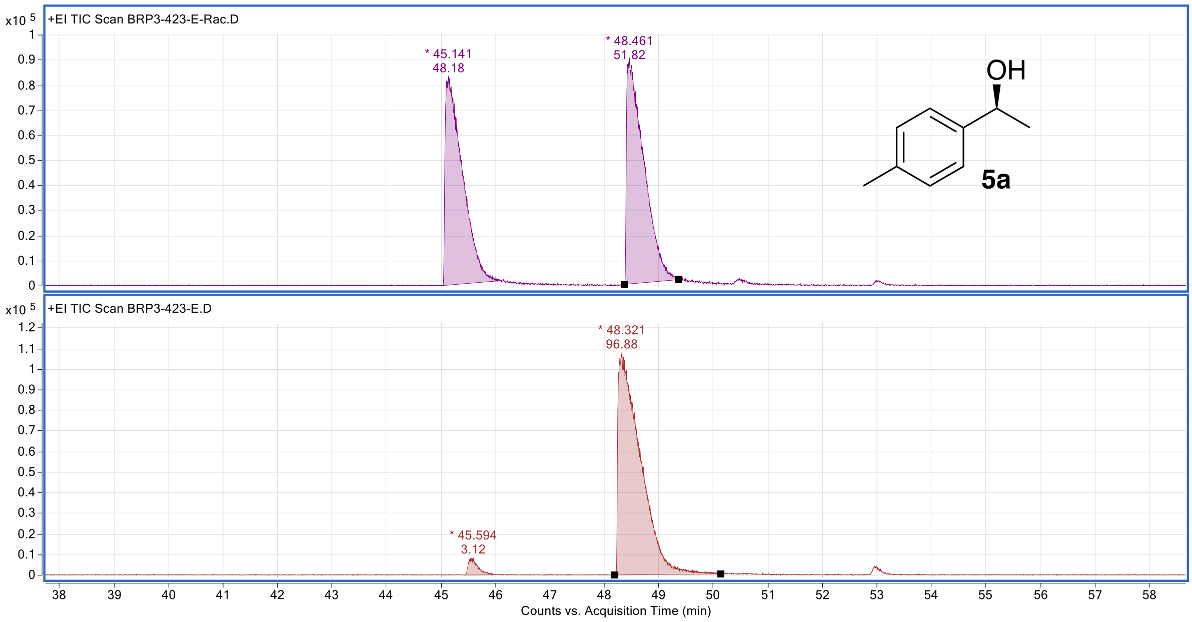
+EI TIC Scan BRP3-423-E-Rac.D
* 45.141
48.18
* 48.461
51.82
OH
5a
+EI TIC Scan BRP3-423-E.D
* 48.321
96.88
* 45.594
3.12
Counts vs. Acquisition Time (min)

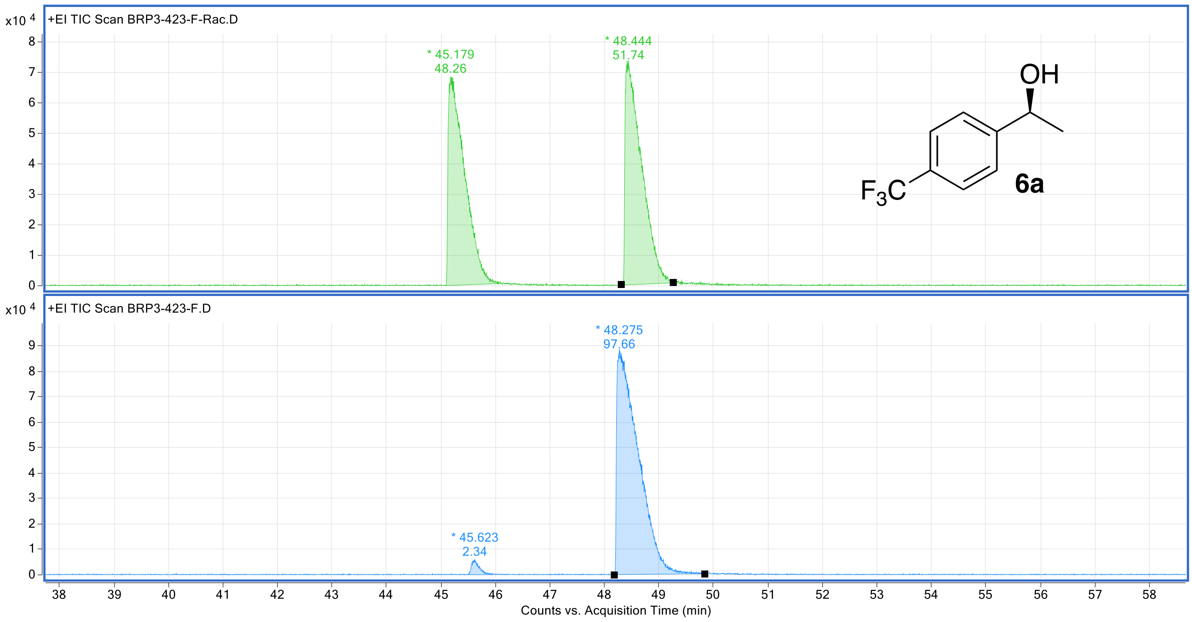
+EI TIC Scan BRP3-423-F-Rac.D
* 45.179
48.26
* 48.444
51.74
OH
F3C
6a
+EI TIC Scan BRP3-423-F.D
* 48.275
97.66
* 45.623
2.34
Counts vs. Acquisition Time (min)

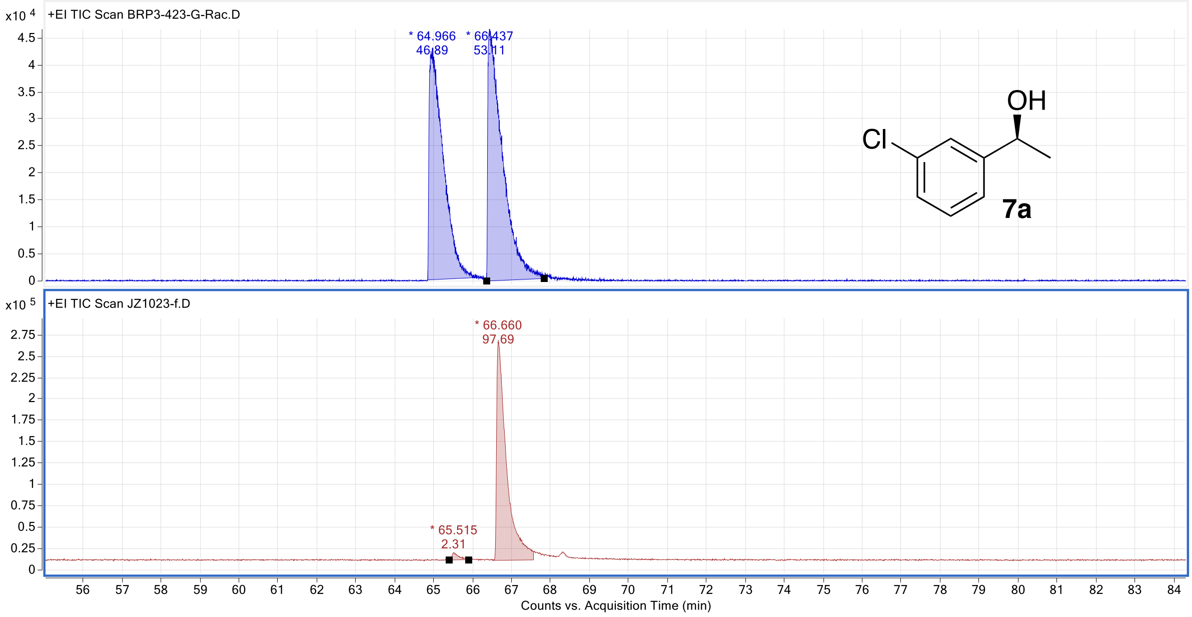
+EI TIC Scan BRP3-423-G-Rac.D
* 64.966
46.89
* 66.437
53.11
OH
Cl
7a
+EI TIC Scan JZ1023-f.D
* 66.660
97.69
* 65.515
2.31
Counts vs. Acquisition Time (min)

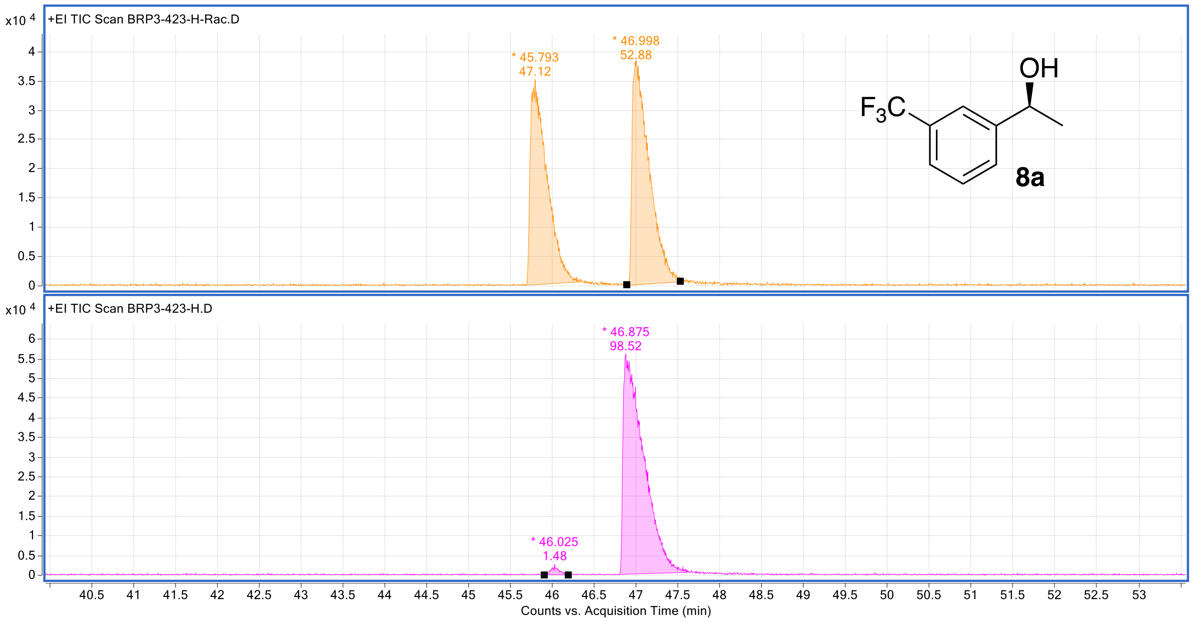
+EI TIC Scan BRP3-423-H-Rac.D
* 45.793
47.12
* 46.998
52.88
F3C
OH
8a
+EI TIC Scan BRP3-423-H.D
* 46.875
98.52
* 46.025
1.48
Counts vs. Acquisition Time (min)

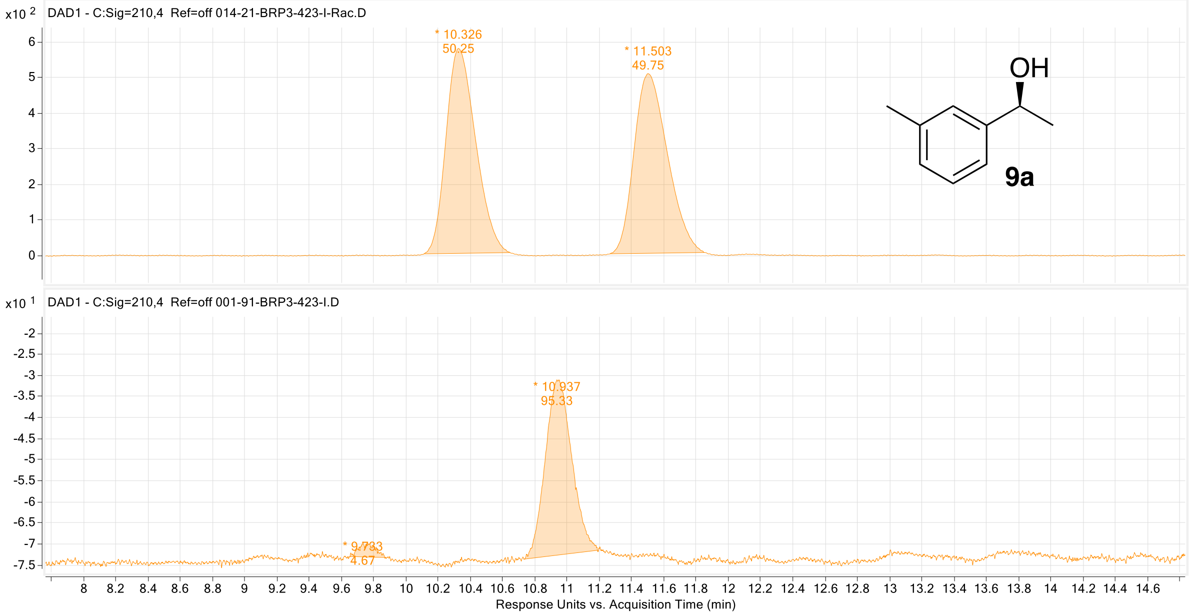
DAD1 - C:Sig=210,4 Ref=off 014-21-BRP3-423-I-Rac.D
* 10.326
50.25
* 11.503
49.75
OH
9a
DAD1 - C:Sig=210,4 Ref=off 001-91-BRP3-423-I.D
* 10.937
95.33
* 9.733
4.67
Response Units vs. Acquisition Time (min)

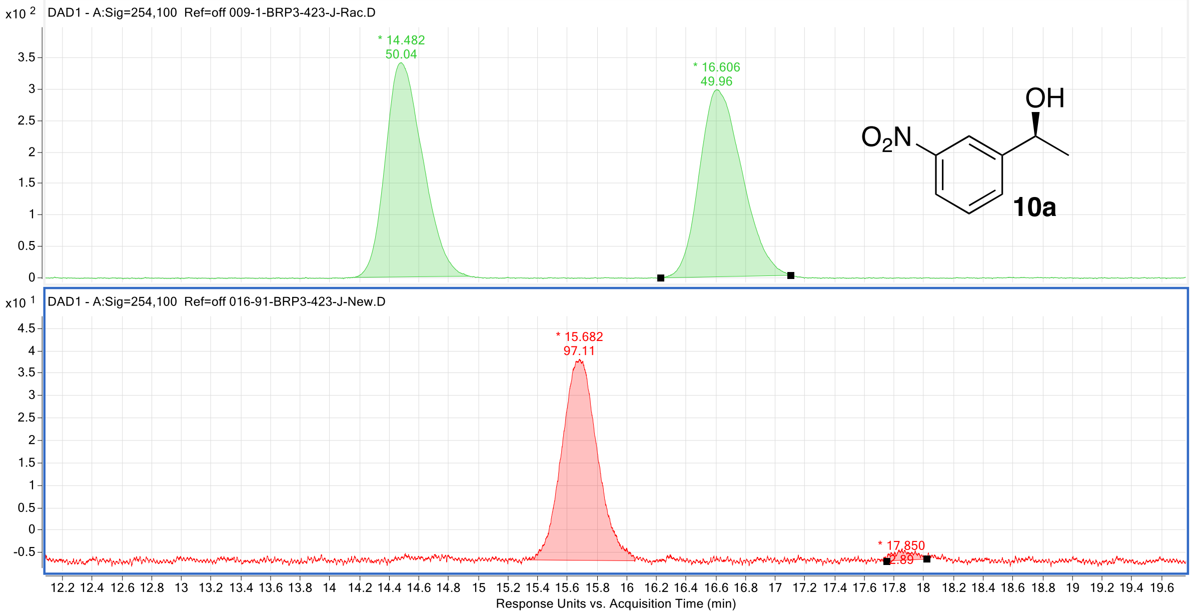
DAD1 - A:Sig=254,100 Ref=off 009-1-BRP3-423-J-Rac.D
* 14.482
50.04
* 16.606
49.96
O2N
OH
10a
DAD1 - A:Sig=254,100 Ref=off 016-91-BRP3-423-J-New.D
* 15.682
97.11
* 17.850
2.89
Response Units vs. Acquisition Time (min)

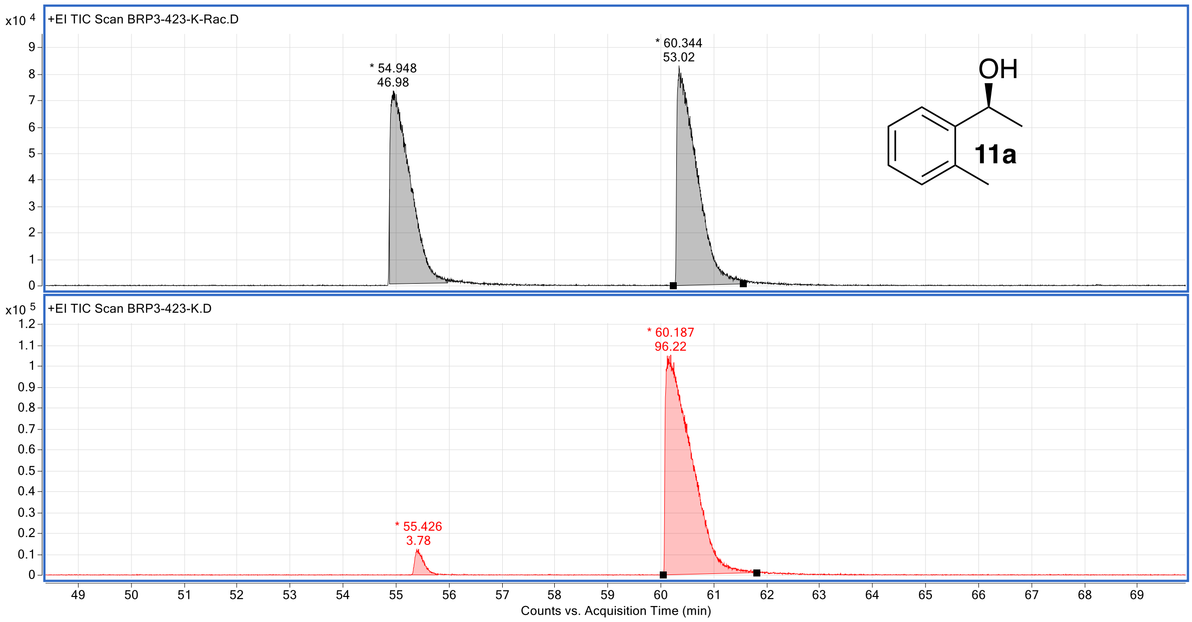
+EI TIC Scan BRP3-423-K-Rac.D
x10 4
* 54.948
46.98
* 60.344
53.02
OH
11a
+EI TIC Scan BRP3-423-K.D
x10 5
* 55.426
3.78
* 60.187
96.22
Counts vs. Acquisition Time (min)

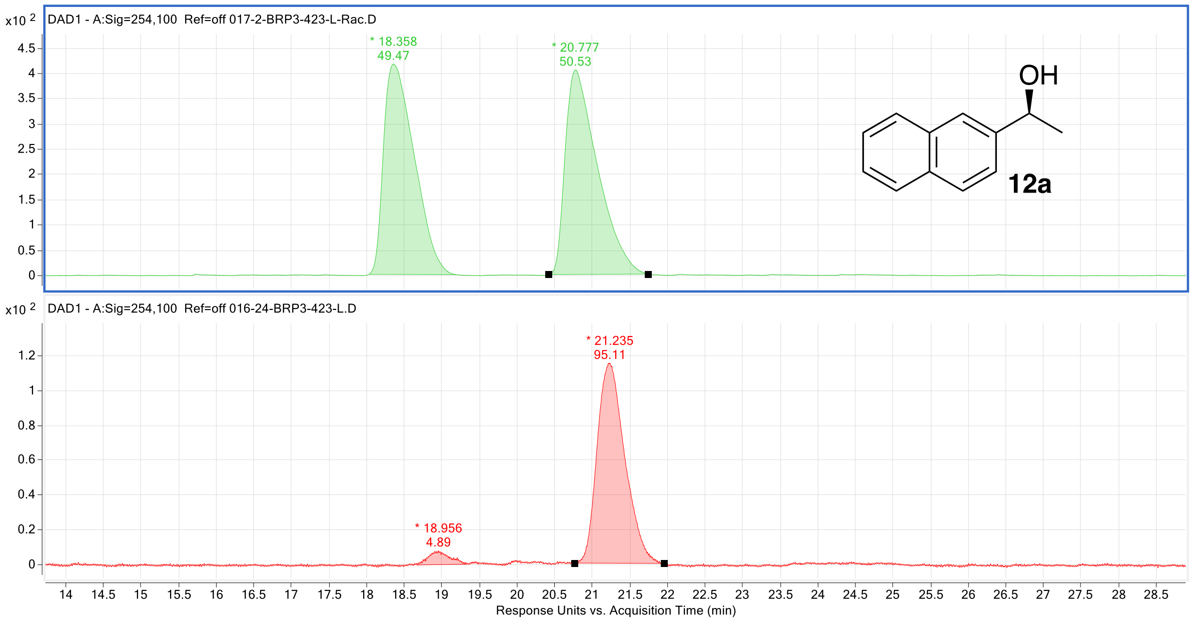
DAD1 - A:Sig=254,100 Ref=off 017-2-BRP3-423-L-Rac.D
x10 2
* 18.358
49.47
* 20.777
50.53
OH
12a
DAD1 - A:Sig=254,100 Ref=off 016-24-BRP3-423-L.D
x10 2
* 18.956
4.89
* 21.235
95.11
Response Units vs. Acquisition Time (min)

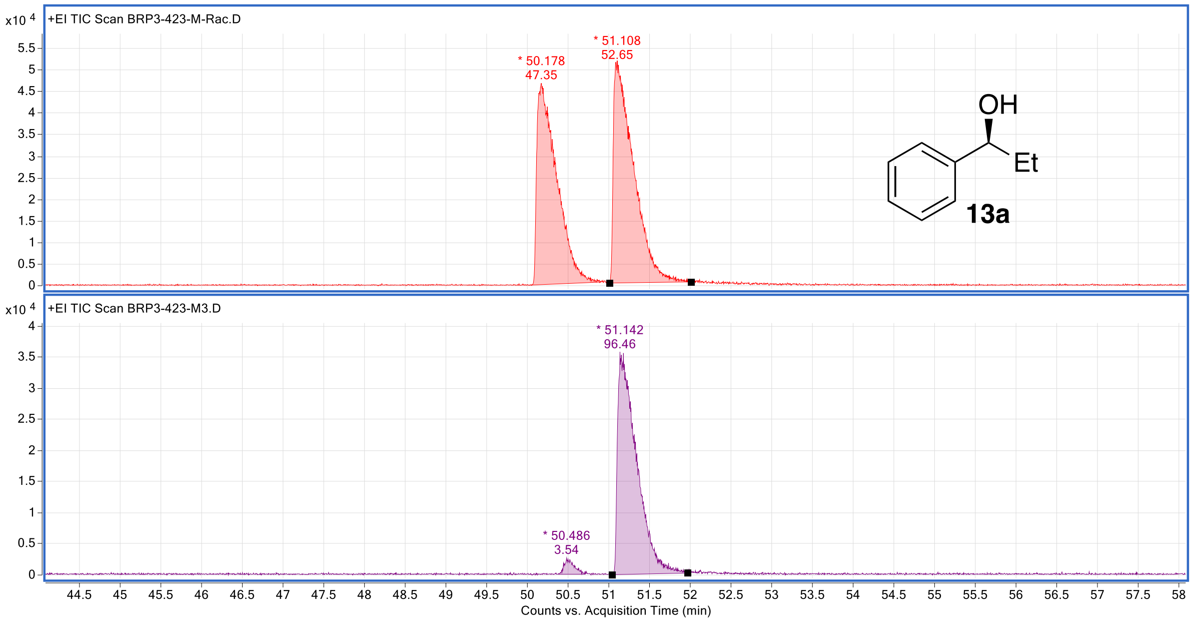
+EI TIC Scan BRP3-423-M-Rac.D
x10 4
* 50.178
47.35
* 51.108
52.65
OH
Et
13a
+EI TIC Scan BRP3-423-M3.D
x10 4
* 50.486
3.54
* 51.142
96.46
Counts vs. Acquisition Time (min)

References

(1) Kerdphon, S.; Quan, X.; Parihar, V. S.; Andersson, P. G. *J. Org. Chem.* **2015**, *80*, 11529-11537.

(2) Li, J-Q.; Andersson, P. G. *Chem. Commun.* **2013**, *49*, 6131-6133.

(3) Quan, X.; Kerdphon, S.; Andersson, P. G. *Chem. Eur. J.* **2015**, *21*, 3576-3579.

(4) Adams, H.; Bawa, R. A.; Jones, S. *Organic & Biomolecular Chemistry* **2006**, *4*, 4206-4213.

(5) Horwell, D. C.; Lewthwaite, R. A.; Pritchard, M. C.; Ratcliffe, G. S.; Ronald Rubin, J. *Tetrahedron* **1998**, *54*, 4591-4606.

(6) Takeuchi, H.; Fujimoto, T.; Hoshino, K.; Motoyoshiya, J.; Kakehi, A.; Yamamoto, I. *J. Org. Chem.* **1998**, *63*, 7172-7179.

(7) Kultyshev, R. G.; Miyazawa, A. *Tetrahedron* **2011**, *67*, 2139-2148.

(8) Toscano, J. P. S., A. D., WO 2014/070919 A l **2014**.

(9) Ji, H.; Li, H.; Martásek, P.; Roman, L. J.; Poulos, T. L.; Silverman, R. B. *J. Med. Chem.* **2009**, *52*, 779-797.

(10) Nanchen, S.; Pfaltz, A. *Helv. Chim. Acta* **2006**, *89*, 1559-1573.

(11) Khan, I. A.; Saxena, A. K. *J. Org. Chem.* **2013**, *78*, 11656-11669.

(12) Huang, J.-D.; Hu, X.-P.; Duan, Z.-C.; Zeng, Q.-H.; Yu, S.-B.; Deng, J.; Wang, D.-Y.; Zheng, Z. *Org. Lett.* **2006**, *8*, 4367-4370.
